# Nanotechnology-mediated strategies for skeletal muscle repair and regeneration: targeted intervention, functional remodeling, and translational challenges

**DOI:** 10.1186/s12951-026-04610-z

**Published:** 2026-05-27

**Authors:** Chengyan Guo, Xianji Wei, Wenjing Li, Guanyi Wang, Binghong Gao, Lingli Zhang, Bo Gao

**Affiliations:** 1 School of Exercise and Health, Shanghai University of Sport, 200438 Shanghai, China; 2 College of Athletic Performance, Shanghai University of Sport, 200438 Shanghai, China; 3 School of Sport, Exercise and Health Sciences, Loughborough University, Loughborough, LE11 3TU UK; 4 Institute of Orthopedic Surgery, Xijing Hospital, Air force Medical University, Xi’an, China; 5 Faculty of Healthy Sciences and Sports, Macao Polytechnic University, 999078 Macao, Macao

**Keywords:** Nanotechnology, Skeletal muscle, Repair, Regeneration, Targeted drug delivery, Biomimetic scaffolds, Nanozymes

## Abstract

Skeletal muscle is an important locomotor and metabolic organ in the human body and plays a central role in maintaining physical activity, energy homeostasis, and structural support. Although skeletal muscle possesses a certain regenerative potential, under pathological conditions such as aging, severe trauma, pathogen infection, myogenic malignancies, and other metabolic diseases, its repair process is often accompanied by impaired myogenic differentiation, inflammatory and oxidative stress imbalance, insufficient vascularization, and limited innervation, ultimately leading to structural muscle damage and functional impairment. These changes not only severely affect patients’ quality of life but also impose a substantial economic burden on healthcare systems, making them an urgent public health issue. Existing therapeutic strategies, including pharmacological treatment, physical interventions, and surgical procedures, can alleviate symptoms to some extent. However, they generally remain limited by insufficient targeting, restricted local bioavailability, and unstable long-term efficacy. In recent years, nanotechnology has shown considerable application potential in skeletal muscle regeneration and related disease intervention, owing to its tunable physicochemical properties, delivery advantages, and tissue-biomimetic capabilities. Based on the specific pathological characteristics of skeletal muscle diseases, this Review systematically summarizes recent advances in nanotechnology, including drug delivery, scaffold construction, nanozyme design, and biomimetic tissue engineering. Particular emphasis is placed on their potential roles in promoting myogenic differentiation, alleviating inflammation and oxidative stress, combating infection and myogenic malignancies, supporting vascularization, and facilitating the recovery of neural structure and function. In addition, this Review further analyzes the clinical challenges and future directions of nanotechnology in the field of skeletal muscle diseases, aiming to provide a reference for related research and the optimization of therapeutic strategies for skeletal muscle disorders.

**Graphical Abstract:**

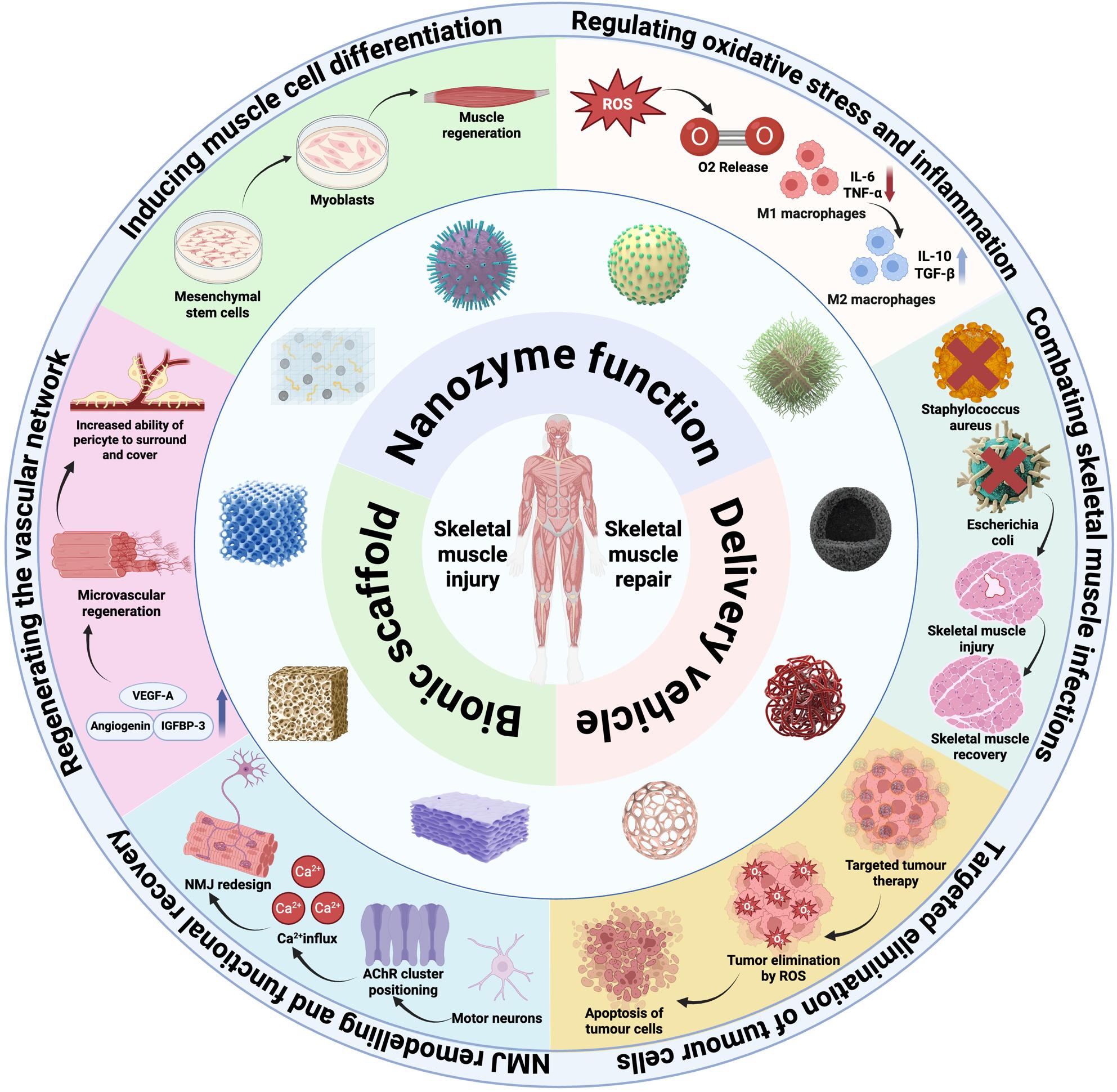

## Introduction

Skeletal muscle is one of the largest organ systems in the human body, accounting for approximately 40% of total body mass, and plays essential roles in locomotion, metabolic regulation, and structural support [1]. Under normal physiological conditions, skeletal muscle tissue possesses significant repair and regenerative potential. For example, when stimulated by mild injury signals, its intrinsic repair mechanisms can be rapidly activated and restore tissue structure and function through a series of highly coordinated molecular and cellular biological processes, thereby effectively responding to the pathophysiological changes caused by injury [2]. However, under pathological conditions such as aging, severe trauma, pathogen infection, metabolic abnormalities, and myogenic malignancies, skeletal muscle homeostasis and regeneration are usually significantly disrupted, ultimately leading to reduced muscle mass, destruction of tissue structure, and impaired functional responses. From the perspective of pathological mechanisms, the disruption of homeostasis caused by skeletal muscle-related diseases and severe injury is not regulated by a single factor. Instead, it involves structural and functional impairments mediated by multiple microenvironmental and biological processes, including restricted myogenic differentiation of satellite cells and myoblasts, imbalance of inflammatory and oxidative stress microenvironments, infection-related tissue destruction, local tissue integrity damage caused by myogenic malignancies, insufficient vascularization, and limited innervation [2–4]. These pathological processes are intertwined and collectively determine the quality of skeletal muscle regeneration and the final outcome of functional reconstruction [5, 6]. Therefore, conducting hierarchical and targeted interventions against the above key barriers is a fundamental prerequisite for improving the efficiency of skeletal muscle repair and optimizing clinical therapeutic outcomes.

Currently, the most common therapeutic strategies for skeletal muscle-related diseases include physical therapy, pharmacological treatment, and surgical intervention [7]. Physical therapy plays an important role in the rehabilitation of skeletal muscle diseases, with a broad range of applications and diverse treatment modalities, including ultrasound therapy, transcutaneous electrical nerve stimulation, extracorporeal shock wave therapy, low-intensity laser therapy, and manual therapy. Therapists can develop individualized treatment plans according to the disease condition and patient-specific factors [8]. However, as a conventional non-invasive intervention, physical therapy still lacks fully clarified standardized treatment protocols and molecular mechanisms, which may impose certain limitations on its clinical application [9]. Pharmacological treatment, such as anti-inflammatory drugs and growth factors, can improve local pathological responses to some extent, but its therapeutic efficacy is often limited by low in vivo delivery efficiency, unstable release kinetics, insufficient tissue targeting, and systemic side effects [10]. Although surgical repair can directly address structural tissue defects, its clinical application remains constrained by surgical trauma, limited availability of donor tissues, incomplete functional recovery, and potential complications [11]. Therefore, developing an efficient and safe therapeutic strategy to enhance the regeneration and repair efficiency of muscle tissue has become a key issue that urgently needs to be addressed in the field of skeletal muscle repair therapy.

In recent years, nanomaterials have shown important potential in the treatment and intervention of skeletal muscle diseases due to their unique physicochemical properties (such as high specific surface area, controlled release efficiency, and surface functionalization capabilities) and biological effects (including enhanced drug delivery, modulated cell behaviour, and improved microenvironment) [12]. Nanomaterials can be used as delivery carriers for the efficient loading of drugs, genes, or growth factors, thereby achieving local delivery and controllable release of the loaded substances [13]. In addition, nanoscaffolds with biomimetic properties can mimic the structural characteristics of the extracellular matrix and provide physical and biochemical support for myocyte adhesion, migration, oriented arrangement, and myogenic differentiation [14, 15]. Moreover, nanomaterials with enzyme-like catalytic activity can participate in local redox regulation and show application potential in anti-infective intervention, intervention against myogenic malignancies, and microenvironmental remodeling [16]. Based on the multifunctional properties of nanomaterials, these materials can act through multiple routes, including the induction of myogenic differentiation, regulation of inflammation and oxidative stress, intervention against infection and myogenic malignancies, vascularization, and reconstruction of neural function, thereby synergistically optimizing the pathological microenvironment of skeletal muscle and accelerating tissue repair [17].

## Pathogenesis and therapeutic strategies for skeletal muscle disorders

### Cellular differentiation dysfunction: myogenic induction of stem cells and myoblasts

Skeletal muscle tissue possesses a remarkable capacity for regeneration, a characteristic that relies primarily on muscle satellite cells (MuSCs), which are located between the basal lamina of muscle fibers and the sarcolemma [18]. Under normal physiological conditions, once injury signals are triggered in skeletal muscle, quiescent MuSCs can be rapidly activated and undergo asymmetric division. One population undergoes self-renewal to maintain muscle cell homeostasis, whereas the other differentiates into myoblasts, which subsequently fuse to form nascent myotubes and further develop into mature myofibers, thereby enabling the reconstruction of skeletal muscle structure and function [19]. However, under pathological conditions such as primary skeletal muscle diseases or severe trauma, the self-repair and compensatory mechanisms of skeletal muscle are severely impaired, making it difficult to achieve structural and functional reconstruction [20]. For example, in Duchenne muscular dystrophy (DMD) muscle, mutations in the DMD gene located in the Xp21.2 chromosomal region lead to the loss or abnormal expression of dystrophin [21], which in turn causes structural and functional abnormalities of the dystrophin-associated glycoprotein complex (DGC), ultimately affecting the mechanical stability of the muscle fiber membrane as well as the myogenic differentiation of MuSCs [22–24]. In recent years, gene therapy has demonstrated potential therapeutic value in the field of skeletal muscle disorders. Its fundamental principle lies in correcting disease-related defects at the molecular level by delivering functional genes, repairing pathogenic mutations or restoring the expression of key proteins, thereby improving muscle fiber stability and slowing disease progression [25]. Consequently, for Duchenne muscular dystrophy (DMD), restoring the expression of dystrophin is the primary therapeutic objective. However, due to the excessive length of the DMD gene cDNA (approximately 14 kb), which significantly exceeds the loading capacity of commonly used viral vectors (such as adeno-associated virus, AAV), full-length gene replacement has long faced significant obstacles in clinical translation [26]. To overcome this limitation, researchers have further developed mini- or micro-dystrophin constructs that retain key functional domains, so as to be compatible with existing delivery systems. For example, Mendell et al. [27] used rAAVrh74 to deliver a codon-optimized human micro-dystrophin transgene driven by the MHCK7 promoter, which resulted in increased micro-dystrophin expression and correct localization in the skeletal muscle of patients with DMD, accompanied by reduced serum creatine kinase levels and improved North Star Ambulatory Assessment scores, suggesting that this strategy has certain therapeutic potential. However, the long-term efficacy and safety of this strategy still require further follow-up and evaluation [27, 28].

In addition to directly correcting primary genetic defects, interventions targeting key cellular components involved in skeletal muscle regeneration and their associated signalling networks have gradually emerged as complementary therapeutic approaches of increasing interest. As the core stem cells responsible for skeletal muscle regeneration, MuSCs are tightly regulated in their activation, proliferation, and differentiation by multiple key signalling pathways, including Notch, PI3K/Akt, AMPK/PGC-1α, Wnt/β-catenin, and TGF-β/Smad [29]. By using exogenous mediators, such as specific pharmacological compounds, bioactive factors, or signalling molecules, targeted modulation of the above pathways can influence the fate determination of MuSCs and promote their commitment to the myogenic lineage. Meanwhile, the proliferation, differentiation, and fusion of myoblasts directly determine myotube formation, the maturation of regenerated myofibers, and ultimately functional reconstruction, and therefore also represent key regulatory targets in skeletal muscle regenerative interventions [30]. In summary, the precise regulation of key signalling pathways within MuSCs and myoblasts provides an important theoretical foundation and potential therapeutic direction for the regeneration and repair of skeletal muscle disorders, as well as for clinical interventions.

### Abnormal microenvironment: targeted regulation of inflammation and oxidative stress

The functional activity of skeletal muscle involves critical immunoregulatory and redox responses [31]. Skeletal muscle atrophy or mass decline is frequently associated with the production of pro-inflammatory cytokines within the tissue [32]. For instance, elevated levels of inflammatory cytokines in skeletal muscle have been observed in patients with muscular atrophy and in animal models induced by sepsis, cachexia, diabetes, or ageing, indicating the detrimental role of inflammation in advancing skeletal muscle pathology [33–35]. Specifically, during the skeletal muscle inflammatory response, nitric oxide synthase (NOS) can rapidly respond to inflammatory stimuli and, through autocrine signaling, promote the production of cytokines and nitric oxide (NO) [36]. At the same time, multiple Toll-like receptors (TLRs) expressed on the surface of skeletal muscle cells are activated by their respective ligands, thereby initiating downstream phosphorylation cascades and inducing the activation of inflammation-related signaling pathways such as NF-κB [37]. These inflammatory signals further promote the sustained release of pro-inflammatory cytokines, including TNF-α, IL-1β, and IL-6, and upregulate the expression of muscle-specific E3 ubiquitin ligases such as MuRF1 and Atrogin-1/MAFbx, thereby enhancing protein degradation mediated by the ubiquitin-proteasome system (UPS) and the autophagy-lysosome system (ALS), ultimately promoting catabolic metabolism in skeletal muscle [38, 39]. Further studies have shown that, in a lipopolysaccharide (LPS)-induced mouse model of muscle atrophy, the expression levels of TNF-α and IL-1 in skeletal muscle are significantly increased and are accompanied by suppression of Akt/mTOR signaling. As the Akt/mTOR pathway represents a major anabolic axis required for maintaining protein synthesis and cellular growth in skeletal muscle, its reduced activity can further inhibit the phosphorylation of key downstream translational regulators, including p70S6K and 4E-BP1, thereby impairing protein translation and synthesis and compromising cell proliferation and myogenic differentiation [40–42]. In summary, the pro-inflammatory response in skeletal muscle not only promotes protein degradation by activating catabolic pathways, but also impairs protein synthesis and regeneration by inhibiting anabolic pathways. Therefore, appropriate regulation of the inflammatory response in skeletal muscle is crucial for establishing a stable cellular microenvironment and promoting effective regeneration.

Macrophages constitute the most abundant inflammatory cell population within the muscle pathological environment, influencing disease progression by modulating their own polarisation and secretion of inflammatory cytokines [43]. Macrophages are broadly categorised into two phenotypes: M1 pro-inflammatory and M2 anti-inflammatory/reparative [44]. M1 macrophages are characterised by high expression of pro-inflammatory cytokines (e.g., IL-1β, TNF-α, IL-6, IL-18), whose activation exacerbates inflammatory responses and accelerates pathological progression in skeletal muscle injury [45]. Conversely, M2 macrophages secrete anti-inflammatory factors (e.g., IL-10) and express characteristic surface markers (CD206, CD163). They not only antagonise pro-inflammatory factors but also enhance tissue regeneration by regulating myogenic differentiation of satellite cells [46–48]. In skeletal muscle inflammatory responses induced by minor exogenous injury, endogenous regulatory mechanisms promote macrophage phenotypic switching from M1 to M2. This dynamic polarisation process establishes the essential microenvironmental foundation for subsequent tissue remodelling and regeneration [49]. However, in chronic skeletal muscle pathologies, the self-regulatory capacity of macrophages is markedly impaired, rendering endogenous polarisation mechanisms insufficient for tissue repair [50]. Consequently, intervention strategies targeting macrophages are of great significance for alleviating the inflammatory response in skeletal muscle.

Maintaining redox homeostasis in skeletal muscle is equally critical for treating skeletal muscle disorders. Within physiological concentration ranges, reactive oxygen species (ROS) function as second messenger molecules, activating satellite cells via redox signaling pathways to initiate skeletal muscle regeneration [51]. However, when ROS levels exceed physiological thresholds, intracellular oxidative stress ensues, leading to persistent ROS accumulation and skeletal muscle dysfunction [52]. Tetrandrine-mediated elevation of ROS levels targets the FoxO3/AKT signalling pathway, promoting ubiquitin-proteasome expression and autophagy processes. This subsequently induces skeletal muscle proteolysis and accelerates muscle atrophy [53]. Furthermore, elevated ROS levels can alter the differentiation fate of satellite cells. Pathological ROS elevation significantly upregulates PRDM12 (PR/SET domain 12) transcription factor expression within satellite cells, promoting the activation of their adipogenic differentiation potential while suppressing myogenic differentiation. This ultimately results in skeletal muscle regenerative impairment and tissue structural disruption [54]. Currently, antioxidant agents demonstrate considerable efficacy in scavenging cellular ROS and alleviating oxidative stress. Nevertheless, enhancing therapeutic precision remains crucial for restoring skeletal muscle homeostasis and promoting tissue regeneration.

### Pathogen invasion: bactericidal anti-infective protection

Under normal physiological conditions, skeletal muscle possesses inherent resistance to bacterial and fungal pathogens. However, injury, surgery, ischaemia, and compromised immune function increase susceptibility to bacterial invasion and infection [55, 56]. Infectious myositis typically arises from contiguous spread from adjacent infection sites, penetrating injuries, ischaemia, foreign bodies, or haematogenous dissemination [57]. Pathogenic bacterial invasion (e.g., Gram-positive, Gram-negative, anaerobic bacteria, mycobacteria, and certain atypical bacteria) induces secretion of adhesins, cytotoxins, superantigens, and immunomodulatory proteins, accelerating skeletal muscle inflammation and necrosis [57, 58]. Staphylococcus aureus, a Gram-positive bacterium, can mediate myositis of varying severity, categorised by inflammatory phase into acute, subacute, and malignant myositis [59]. Its core pathological mechanism involves the abnormal activation and recruitment of antigen-specific CD8⁺ cytotoxic T cells around muscle fibers. These effector T cells recognise muscle fibers expressing major histocompatibility complex class I molecules via their TCR receptors, subsequently invading and releasing cytotoxic mediators that induce myocyte apoptosis and fibronecrosis [60]. Furthermore, Pseudomonas aeruginosa, a Gram-negative pathogen, represents a significant causative agent in acquired infections [61]. Infected skeletal muscle secretes 2-aminoacetophenone, leading to abnormal expression of NADPH oxidase 2 (NOX2) and consequent excessive ROS production. Notably, abnormal ROS accumulation within skeletal muscle can directly or indirectly suppress antioxidant enzyme activity (including superoxide dismutase, catalase, and glutathione peroxidase), exacerbating tissue oxidative stress levels and promoting cellular autophagy and myofibrillar degradation [62]. To achieve antimicrobial therapy in skeletal muscle, early antibiotic administration may appropriately reduce pathogen activity and delay disease progression. However, excessive therapeutic doses or prolonged use can lead to adverse effects such as increased drug resistance, microenvironmental disruption, and multi-organ toxicity [63]. Consequently, developing locally specific targeted delivery systems for efficient pathogen clearance holds critical therapeutic significance for promoting skeletal muscle functional recovery and preventing secondary systemic infections.

### Rhabdomyosarcoma: precision local intervention in a malignant skeletal muscle pathological context

Rhabdomyosarcoma (RMS) is a special malignant pathological entity within the spectrum of skeletal muscle diseases and is also one of the most common soft tissue sarcomas in children [64]. Although RMS exhibits differentiation characteristics of the skeletal muscle lineage, its aetiology does not stem from simple regenerative failure following damage to mature muscle fibers; rather, it is primarily attributed to the malignant transformation of primitive mesenchymal cells or myogenic progenitor cells possessing myogenic differentiation potential [65, 66]. At the molecular level, certain subtypes of RMS (such as alveolar RMS) typically involve PAX3-FOXO1 or PAX7-FOXO1 fusion genes as key molecular events [67]. Furthermore, the abnormal activation of the insulin-like growth factor (IGF) axis and pro-proliferative and anti-apoptotic signalling pathways such as PI3K-AKT-mTOR can directly enhance tumour cell proliferation, anti-apoptotic properties and differentiation arrest; these mechanisms act synergistically to drive the malignant progression of RMS [68, 69]. As the pathological process progresses, the malignant pathological microenvironment induced by RMS can markedly impair the normal reparative capacity and functional maintenance of muscle tissue. Specifically, RMS not only directly disrupts skeletal muscle structural integrity through the sustained increase in local tumor burden, but also aggravates muscular structural damage through infiltrative tumor growth, space-occupying compression, and treatment-related injury [70, 71]. Meanwhile, intrinsic inflammatory responses within the tumor microenvironment, abnormally elevated oxidative stress, and local homeostatic imbalance further exacerbate injury to the surrounding tissues and directly compromise the structural stability and functional reserve of skeletal muscle [72, 73]. Therefore, RMS can be regarded as a special pathological context of skeletal muscle disease driven primarily by malignant lesions and characterized by local structural tissue destruction together with persistent deterioration of the microenvironment. Current clinical treatment strategies for RMS primarily rely on conventional chemotherapy (such as vincristine, actinomycin D and cyclophosphamide/ifosfamide), radiotherapy and surgical resection [74–76]. Although the aforementioned regimens have demonstrated some efficacy in controlling the progression of RMS, their overall therapeutic efficacy remains limited by issues such as insufficient local targeting, significant systemic toxicity, uneven distribution of the drug within the tumour, and a high likelihood of resistance developing [77]. In this context, nanodelivery systems can be conjugated with targeting peptides or other functional ligands to improve the accumulation and cellular uptake of therapeutic agents at tumor sites, thereby enhancing local antitumor efficacy while reducing off-target organ exposure [78]. In addition, responsive delivery strategies based on tumor microenvironmental characteristics or exogenous physical stimuli may enable more precise local triggered release of drugs at lesion sites, thereby further improving the spatiotemporal controllability of the delivery process [79]. Consequently, given the specific pathological characteristics of RMS, the potential value of nanotechnology lies primarily in improving the precision of local intervention, reducing toxic side effects, and minimizing damage to the structure and function of the surrounding skeletal muscle tissue.

### Angiogenesis impairment: vascular integration and support

Angiogenesis underpins embryonic development and organogenesis while also forming the basis for pathophysiology in numerous diseases, including diabetes, Duchenne muscular dystrophy, and individual aging [80, 81]. Skeletal muscle capillaries serve as transporters of nutrients, oxygen, and various metabolites, enhancing muscle protein synthesis by ensuring the delivery of amino acids and growth factors to muscle fibers [82]. Thus, a close structural and functional relationship exists between muscle fibers and capillaries. In aging populations, reduced skeletal muscle capillary density impairs satellite cell differentiation, potentially representing a key factor in muscle atrophy, quality decline, and progressive functional loss [83]. In type 2 diabetes mellitus (T2DM) patients, altered skeletal muscle function and insulin resistance are highly correlated with significantly reduced skeletal muscle capillary density, potentially due to decreased vascular endothelial growth factor (VEGF) expression [84, 85]. Furthermore, DMD mice exhibit severe vascular density reduction and markedly impaired angiogenesis [86, 87]. Exercise not only delays age-related muscle loss but also enhances oxygen and nutrient transport efficiency by promoting capillary neogenesis within skeletal muscle and expanding the vascular-muscle fiber exchange interface [88]. Moreover, exercise upregulates protein synthesis rates, induces myocyte proliferation and differentiation, ultimately achieving positive feedback regulation of skeletal muscle structure and function [89]. Although extensive evidence supports exercise therapy’s beneficial effects on the skeletal muscle system, its clinical application remains subject to population heterogeneity [90]. Certain special populations (e.g., patients with severe osteoporosis, advanced joint diseases, or specific metabolic/neuromuscular disorders) require individualized risk assessment and tailored exercise prescriptions [91]. Leveraging the theoretical framework and technological systems of nanomedicine, the design and construction of functional skeletal muscle-targeted delivery systems and bioengineered scaffolds can effectively promote local tissue angiogenesis, offering promise for achieving highly vascularized functional tissue integration.

### Imbalanced innervation: structural and functional reconstruction of the neuromuscular junction

**Fig. 1:**
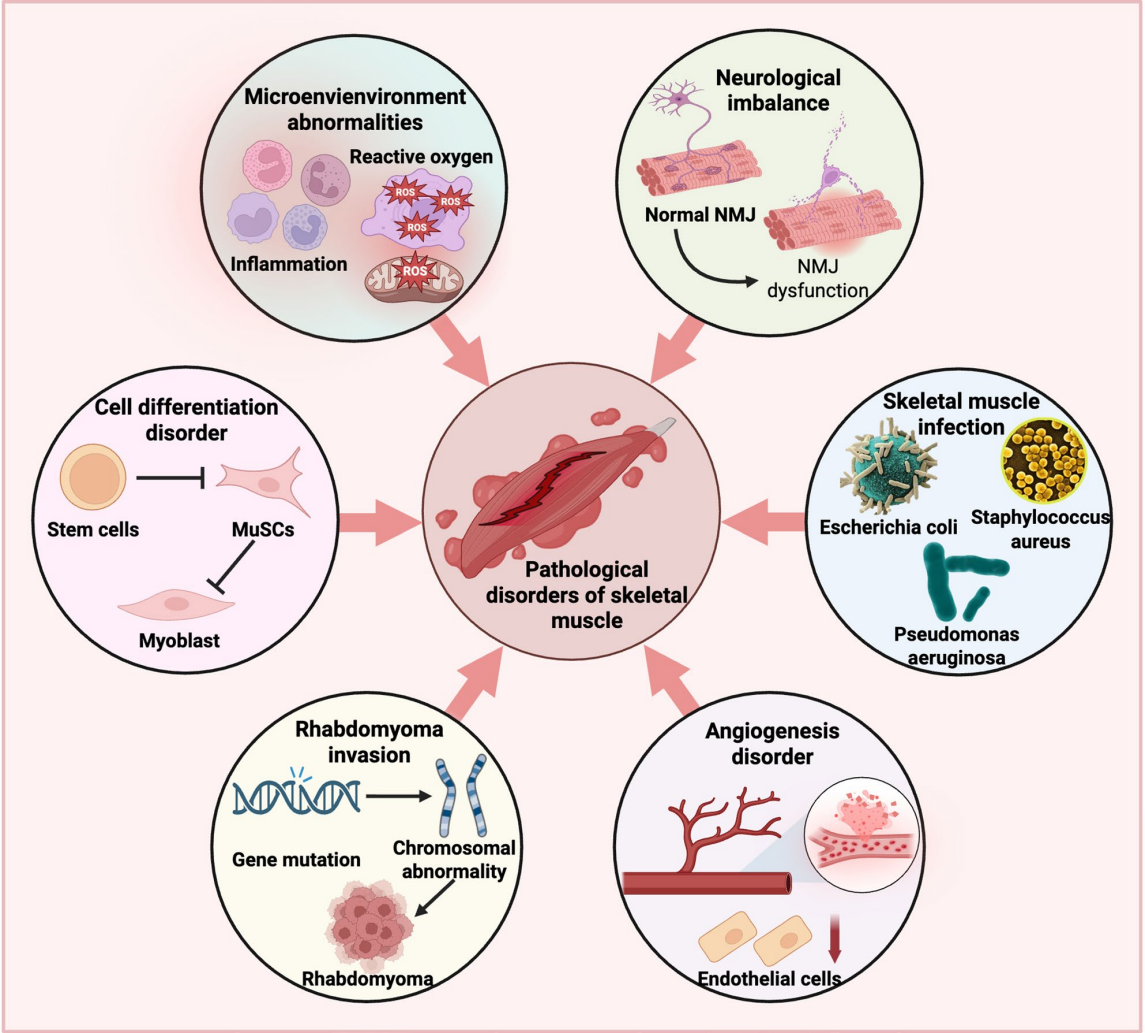
The pathogenesis and multidimensional therapeutic targets of skeletal muscle disorders. The pathogenesis of skeletal muscle diseases is regulated by multiple factors, including myocyte differentiation dysfunction, inflammatory responses and oxidative stress, pathogen infection, tumor cell infiltration and proliferation, impaired vascular network formation, as well as neuromuscular innervation impairment. Targeted intervention against these key pathological processes has become an important therapeutic strategy for skeletal muscle disorders

During skeletal muscle regeneration and remodeling, the microvascular supply system provides the transport support required for the delivery of oxygen and nutrients to the injured region, whereas the reconstruction of innervation is a key step determining whether newly formed myofibers can achieve functional recovery [92]. The neuromuscular junction (NMJ) is a highly specialized synaptic structure between motor nerve terminals and muscle fibers, and its structural integrity is important for maintaining myofiber homeostasis, excitation–contraction coupling, and muscle function [93]. However, factors such as trauma, peripheral nerve injury, aging, and long-term disuse can all lead to skeletal muscle denervation, thereby inducing degenerative changes in NMJ structure and function, which manifest as myofiber atrophy, structural disorganization, and impaired functional responses [94]. Notably, postsynaptic acetylcholine receptors (AChRs), as the principal receptor apparatus in the NMJ endplate region, require ordered clustering not only to constitute the basis of postsynaptic structural stability, but also to serve as a prerequisite for efficient neural impulse transmission across the muscle fiber surface [95]. Specifically, NMJ degeneration caused by skeletal muscle denervation can manifest as fragmentation, dispersion, and abnormal spatial distribution of AChR clusters, which further weakens effective neural impulse transmission in muscle fibers and affects the maintenance and maturation of the endplate structure [96]. At the molecular level, structural homeostasis of the NMJ endplate region mainly depends on the high-density and spatially specific clustering of AChRs. This process is primarily mediated by activation of the agrin–LRP4–MuSK signaling axis with the involvement of rapsyn, ultimately completing the assembly and stable maintenance of AChR clusters [97]. Therefore, destabilization of AChR clustering is not merely an accompanying phenomenon after denervation, but an early key event that impairs NMJ reconstruction and limits functional recovery. In summary, skeletal muscle dysfunction caused by denervation is essentially closely associated with destabilized AChR clustering and impaired endplate assembly. Therefore, targeting the specific and ordered clustering of AChRs in the endplate region and maintaining the stability of these clusters may represent an important interventional target for restoring NMJ structural integrity, improving reinnervation efficiency, and promoting the transition of skeletal muscle from structural repair toward functional recovery and maturation [98] (Fig. 1).

## Biomedical functions of nanomaterials

Based on the pathological barriers and therapeutic demands described above, exogenous drugs and bioactive factors are inevitably subject to clearance and degradation by the innate immune system during treatment. They often show poor targeting efficiency and low specificity, making it difficult to achieve effective therapeutic concentrations in target tissues [99]. The development of nanotechnology has provided a potential approach to overcoming these limitations. This technology not only helps improve the local delivery efficiency and bioavailability of therapeutic molecules, but also contributes to the regulation of cellular behavior, microenvironmental remodeling, and support for tissue repair [100]. Therefore, nanomaterials have gradually become an important functional platform in skeletal muscle repair and regeneration research [101]. According to the strict definition proposed by the National Nanotechnology Initiative, nanomaterials refer to materials with a size range of 1–100 nm in at least one dimension [102]. These materials can be composed of structural elements such as single crystals, polycrystals, or molecules. According to their material composition and chemical constituents, they can be broadly classified as organic or inorganic materials [103]. Due to their unique quantum size effects, surface effects and confinement effects, nanomaterials exhibit physical and chemical properties that are markedly different from those of conventional materials [104]. Currently, nanomaterials have found widespread application in numerous cutting-edge fields within biomedicine, encompassing key areas such as drug delivery systems, tissue engineering, disease prevention and control, diagnostic and therapeutic imaging, and multimodal synergistic therapy [105].

### Drug delivery: targeted delivery and precise controlled release

With their exceptionally high porosity and enormous specific surface area, nanomaterials offer significant advantages for drugs and other active substances [106]. In drug delivery, nanomaterials not only enhance encapsulation and delivery efficiency for conventional drugs but also efficiently carry short-half-life gene-based therapeutics (including siRNA, miRNA, lncRNA, and plasmid DNA), enabling precise regulation of specific genes [107, 108]. Furthermore, nanodelivery systems demonstrate exceptional potential for encapsulating and delivering hormonal drugs, bioactive factors, and even stem cells [109]. However, as exogenous particles, nanomaterials inevitably encounter the body’s immune rejection response. Macrophages within the microenvironment rapidly detect and phagocytose these foreign substances, leading to reduced therapeutic efficacy and systemic inflammatory reactions [110]. To overcome such biological barriers, surface-modified functionalized nanomaterials—such as polyethylene glycol (PEG) coating or stem cell membrane encapsulation—can significantly enhance carrier biocompatibility and circulation survival rates [111]. Furthermore, rational design of the physicochemical properties of nanocarriers (e.g., size, morphology, and surface charge) can substantially reduce interactions between the material and the immune system [112]. Compared to traditional therapeutic approaches, nanodelivery systems effectively suppress in vivo biodegradation and immune interception of drugs, significantly improving drug loading capacity, integrity, and stability. This provides crucial technical support for efficient, controllable drug intervention [113]. Furthermore, to enhance treatment precision, specific ligands (such as peptides, antibodies, or aptamers) are typically conjugated to accurately identify diseased tissues and achieve localized targeted delivery, minimizing unnecessary accumulation in off-target organs [114].

### Scaffold carrier function: synergistic promotion of skeletal muscle repair through bionic structures and bioactivity

To enhance treatment persistence and biocompatibility, nanoscale scaffolds represent a promising therapeutic approach for volumetric muscle injuries, garnering extensive attention in recent decades [115]. In recent years, nanomaterials have been extensively engineered into biomimetic scaffolds to replicate the in vivo tissue microenvironment, promoting cell adhesion, proliferation, and differentiation [116, 117]. Furthermore, nanoscale scaffolds can serve as reservoirs for various conventional drugs, gene therapies, cytokines, and immunosuppressants, enabling controlled-release regulation of cellular metabolic reprogramming to accelerate skeletal muscle tissue regeneration [118]. In terms of fabrication, nanoscaffolds can be engineered with precise control over their size and morphology through a variety of advanced techniques, including electrospinning, photolithography, ice templating, particulate leaching, template synthesis, thermally induced phase separation, solvent casting, molecular self-assembly, freeze-drying, and three-dimensional bioprinting (3D bioprinting) [119, 120]. Different fabrication methods endow nanoscaffolds with distinct structural characteristics and biological functions, thereby providing a diverse range of engineering strategies for constructing biomimetic microenvironments for skeletal muscle. Among these, scaffold systems capable of controlling fiber orientation and integrating functional properties demonstrate significant practical value in inducing the ordered alignment of muscle cells and promoting their differentiation and functional maturation [119, 121].

Electrospinning stands as the most prevalent method for fabricating fibrous scaffolds in tissue engineering [122]. This technique leverages the electrostatic propulsion of natural or synthetic source polymers within a high-voltage electric field to achieve precise spinning and form continuous fiber structures spanning micrometer to nanometer diameters [123]. Furthermore, by adjusting process parameters such as electric field orientation, intensity, flow rate, and collection methods, nanofiber scaffolds with three-dimensional topological structures can be produced. Compared to two-dimensional scaffolds, these structures significantly enhance the adhesion, proliferation, and oriented alignment of multiple components—including myoblasts, satellite cells, collagen, and growth factors—thereby highly mimicking the complex spatial configurations of muscle fibers, nerves, and vascular networks found in natural skeletal muscle tissue [124]. Thus, electrospun nano-scaffolds hold significant value in integrating advanced regenerative strategies like stem cell therapy and 3D bioprinting, offering feasible engineered solutions for diverse skeletal muscle tissue repair.

Functional skeletal muscle contraction is precisely regulated by electrical signals. Neuromuscular electrical stimulation (NMES), as a non-pharmacological physical intervention, has been widely applied to delay skeletal muscle degenerative diseases [125]. Research indicates that NMES can induce involuntary muscle contractions, thereby enhancing oxidative enzyme activity, promoting capillarization within muscle fibers, and modulating muscle fiber-type transitions. Concurrently, it boosts myocyte proliferation, differentiation capacity, and other biological functions [126]. Therefore, constructing electrically conductive bio-scaffolds holds significant importance for muscle tissue engineering. Conductive nanofiber scaffolds, with electrical conductivity similar to natural skeletal muscle tissue, can transmit endogenous bioelectric signals in situ, significantly enhancing the adhesion, proliferation, and myogenic differentiation of C2C12 myoblasts. They are thus regarded as highly promising candidate materials for muscle engineering scaffolds [127]. However, conductive scaffolds may exhibit certain application limitations due to differing physicochemical properties. For instance, metallic nanomaterials (e.g., gold and silver nanoparticles) possess inherent conductivity but suffer from poor degradability and potential cytotoxicity [128, 129]. Carbon-based nanomaterials (e.g., carbon nanotubes, graphene) offer excellent charge transport capabilities yet face challenges such as low water solubility, complex fabrication processes, and suboptimal biocompatibility [130]. By contrast, conductive polymers, when combined with natural or synthetic polymers, can not only retain favorable electrical conductivity and processability, but also improve the biocompatibility, biodegradability, and cell-adhesive properties of the resulting materials, and have therefore emerged as a more promising class of conductive biomaterials [131, 132]. From the perspective of scaffold morphology, conductive polymer-based constructs for skeletal muscle engineering can be broadly classified into three types: hydrogels, films, and three-dimensional porous scaffolds. Conductive hydrogels are typically composed of highly hydrated polymer matrices, such as gelatin methacryloyl (GelMA) or gelatin, combined with conductive components such as polypyrrole (PPy), thereby forming three-dimensional network structures that integrate hydration, flexibility, and electrical conductivity. Their major advantage lies in their closer resemblance to the native extracellular matrix environment, making them particularly suitable for cell encapsulation, bioprinting, and coupling with electrical stimulation [133, 134]. Conductive films are generally fabricated by combining conductive polymers, such as PPy and polyaniline (PANI), with elastic film-forming materials, thereby generating a surface-controllable two-dimensional continuous interface that is conducive to C2C12 cell adhesion, proliferation, and myogenic differentiation [135]. In contrast, conductive three-dimensional porous scaffolds are typically constructed by integrating conductive polymers with natural or synthetic macromolecules, such as collagen and polycaprolactone (PCL), to form three-dimensional structures with interconnected porosity and mechanical support. These scaffolds are more suitable for the repair of volumetric muscle loss (VML), as they can jointly guide myoblast alignment and myotube formation through the combined effects of a conductive microenvironment, pore architecture, and fiber orientation [136]. In summary, conductive polymer hydrogels, films and three-dimensional porous scaffolds each possess distinct characteristics in terms of composition, structural features and applications, and are gradually emerging as a key direction for the development of conductive scaffolds for skeletal muscle tissue engineering. **(**Table 1**)**

**Table 1:** Representative conductive polymer-based scaffolds for skeletal muscle tissue engineering

Scaffold type	Material composition	Structural feature	Main application	Advantages	Limitations	Ref. No.
Conductive hydrogels	GelMA or gelatin with PPy or other conductive polymers	Hydrated 3D conductive networks	Cell encapsulation, 3D bioprinting, and electrical stimulation-related skeletal muscle engineering	Soft, hydrated, and cell-compatible matrix, easy integration with cells and bioactive molecules, tunable electrical properties	Limited mechanical robustness, uncertain degradation kinetics, long-term conductivity and in vivo muscle specificity require further validation	^[133, 134]^
Conductive films	PPy or PANI with elastic film-forming matrices	2D conductive interfaces with tunable surfaces	C2C12 myoblast culture and in vitro myogenic differentiation models	Uniform conductive surface, easy control of surface properties, high reproducibility for in vitro evaluation	Limited 3D tissue mimicry, insufficient evidence for in vivo integration and off-target distribution	^[135]^
Conductive three-dimensional porous scaffolds	Conductive polymers with collagen, PCL, or other macromolecules	Interconnected porous 3D conductive structures	VML repair and scaffold-based skeletal muscle tissue engineering	Stable three-dimensional support, interconnected pores for cell infiltration and nutrient diffusion, tunable architecture for tissue ingrowth	Complex fabrication, vascularization, innervation, degradation control, and long-term host integration remain unresolved	^[136]^

### Nanozymes: targeted catalytic clearance and metabolic regulation

Natural enzymes serve as key catalysts regulating diverse biological processes within the body, playing vital roles in metabolic regulation, nutrient uptake, energy conversion, and disease onset and progression [137–139]. However, the catalytic activity of natural enzymes is susceptible to various factors such as temperature, pH, and pathological microenvironments, limiting their stability [140]. With the rapid advancement of nanotechnology, nanomaterials have been endowed with enzyme-like catalytic capabilities—termed “nanocatalysts”—due to their high specific surface area, abundant active sites, and functionalizability [141]. Compared to natural enzymes, these materials exhibit high catalytic stability, low manufacturing and storage costs, and flexible preparation processes, demonstrating broad application prospects in disease treatment [142].

In recent years, nanozymes have attracted increasing attention as a non-pharmaceutical intervention strategy [143]. Diverse nanomaterials with enzyme-like catalytic functions have been successfully developed, including oxidase-like (OXD-like), superoxide dismutase-like (SOD-like), catalase-like (CAT-like), and peroxidase-like (POD-like) nanozymes [144]. These materials effectively regulate intracellular redox states and catalyze specific enzymatic reactions, thereby participating in ROS metabolic regulation [145]. Nanozymes with different catalytic types possess distinct application scenarios, while those exhibiting SOD-like, CAT-like, and POD-like activities primarily exert antioxidant functions [146]. For instance, V₂O₅ nanozymes possess intrinsic vanadium haloperoxidase-like and glutathione peroxidase-like activities, which can reduce total intracellular ROS levels by participating in the GSH/GSSG redox cycle, thereby exerting general antioxidant protection. Furthermore, to further enhance the spatial targeting accuracy of nanozymes, the characteristic negative membrane potential of mitochondria can be exploited by conjugating lipophilic cationic moieties such as triphenylphosphonium (TPP), thereby significantly increasing the enrichment of nanozymes in dysfunctional mitochondria [147]. For example, TPP-modified molybdenum disulfide (MoS₂) quantum dots have been shown to enter the cytoplasm following endocytosis, after which some escape retention in lysosomes and localize to dysfunctional mitochondria. This nanosystem possesses both SOD-like and CAT-like enzymatic activities, enabling it to reduce mitochondrial ROS levels and alleviate tissue inflammatory responses [148]. These findings suggest that the focus of nanozyme research has gradually shifted from the broad-spectrum scavenging of intracellular ROS to the localization of nanoparticles in regions adjacent to mitochondria and the regulation of their functions. However, in skeletal muscle tissue, these processes remain primarily constrained by pathological extracellular matrix (ECM) deposition and structural remodeling [149]. A dense ECM may restrict nanoparticle diffusion within the local interstitial space and limit their distribution in proximity to myofibers [150]. At the same time, after cellular uptake, they still need to further escape from the retention in lysosomes before they can effectively approach the oxidative sites related to mitochondria [151, 152]. In addition, direct evidence remains insufficient as to whether nanozymes can achieve precise capture of mitochondria-derived ROS (mROS) within diseased skeletal muscle. This limitation is more likely attributable to the highly confined and complex subcellular microenvironment in which mROS resides [153]. Previous studies have shown that mROS-related signals are characterized by an extremely short half-life and a highly restricted diffusion and effective range, typically described as approximately 10–20 nm [154]. Accordingly, future research should place greater emphasis on improving the localization precision and intervention selectivity of mitochondrial oxidative microdomains, so as to minimize potential adverse effects on skeletal muscle repair and regeneration [155] (Table 2).

**Fig. 2:**
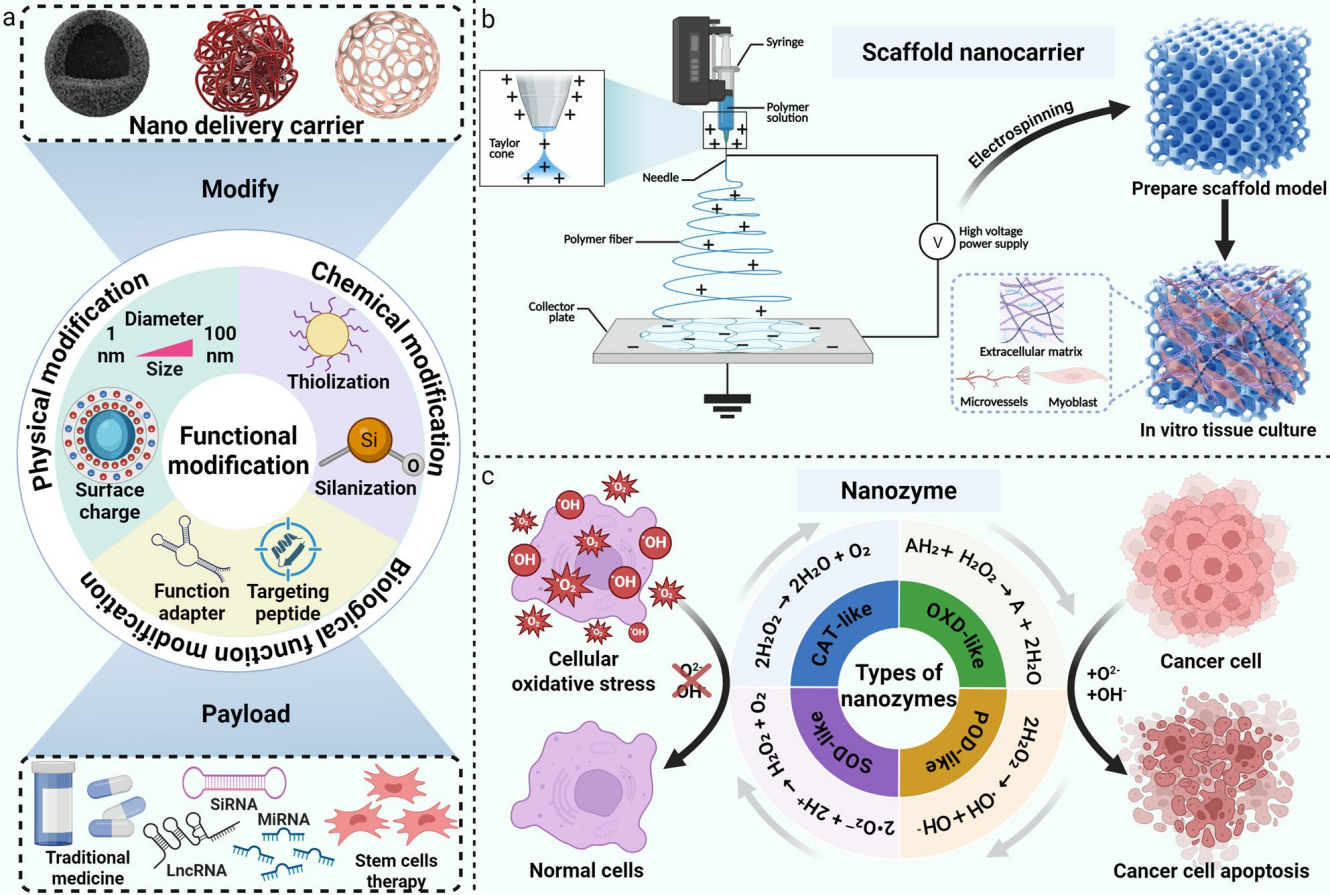
Multifunctional roles of nanomaterials in biomedicine. (**a**) Schematic illustration of engineered nanomaterials serving as versatile biocarriers for the targeted delivery of therapeutic agents, including conventional drugs, microRNAs, and stem cells. (**b**) Construction of biomimetic scaffolds and their application in in vitro skeletal muscle culture and regeneration models. (**c**) Nanozymes with multi-enzyme mimetic activities for ameliorating oxidative stress and exerting antitumor effects

Beyond ROS neutralization, oxidase-like nanozymes can catalyze reactive radical generation for antibacterial and antitumor therapies [156]. Fang et al. [157] leveraged the oxidase-mimetic properties of palladium (Pd) nanozymes to effectively combat both Gram-positive and Gram-negative bacteria. Pd nanozymes exhibit high affinity for O₂, enabling efficient penetration into bacterial cells to induce intracellular ROS and radical production, thereby fully activating their antibacterial effects. This catalytic mechanism is also widely applied in chemodynamic therapy (CDT), where it mediates Fenton-like reactions to trigger toxic radical bursts within tumor cells, inhibiting proliferation and disrupting biological functions [158] (Fig. 2).

**Table 2:** Representative redox-regulatory nanozymes and their potential application scenarios in skeletal muscle repair

Nanozyme type	Representative nanozymes	Core catalytic feature	Potential application scenario	Limitations	Ref. No.
OXD-like nanozymes	Pd, Pt, Au, and metal oxide nanozymes	O₂-driven oxidation and ROS generation	Catalytic antibacterial therapy for infected muscle injury and antitumor intervention in RMS-related pathological contexts	Limited substrate selectivity, excessive oxidative activity, possible persistence of metallic components	^[156, 157, 158, 159, 160]^
SOD-like nanozymes	CeO₂, Mn₃O₄, MoS₂ quantum dots, carbon dots, and graphene-derived nanozymes	O₂•⁻ dismutation into H₂O₂ and O₂	Reduction of intracellular or mitochondrial oxidative stress in damaged muscle cells	Secondary H₂O₂ accumulation, oxidation-state and surface-chemistry dependence, intracellular delivery barriers	^[143, 148, 161, 162]^
CAT-like nanozymes	MnO₂, Pt, MoS₂ quantum dots, and MXene/ MnO₂-based nanocomposites	H₂O₂ decomposition and O₂ generation	Regulation of oxidative stress, hypoxia, and inflammatory microenvironments during skeletal muscle regeneration	Dependence on H₂O₂ availability and local pH, surface passivation in biological fluids, potential metal ion leaching and long-term retention	^[146, 148, 163, 164]^
POD-like nanozymes	Fe₃O₄, Pd, Pt, graphene-derived nanozymes, and MOF-based nanozymes	H₂O₂-driven oxidation and radical generation	Catalytic antibacterial or antitumor therapy in infection- or tumor-associated oxidative microenvironments	Dependence on H₂O₂ and acidic conditions, nonspecific radical toxicity, material stability concerns	^[157, 165, 166, 167, 168]^

## Applications of nanotechnology in skeletal muscle disorders

### Precise induction of skeletal muscle cell differentiation via nanotechnology

Due to their high loading capacity, nanomaterials are commonly employed as drug delivery carriers and nanoscale scaffolds, minimizing drug leakage and enabling precise targeted delivery [169]. The following provides a comprehensive overview of the diverse applications of nanomaterials in inducing myocyte differentiation.

#### Activation of myoblast function and differentiation promotion by nanodelivery systems

Phosphatase and tensin homolog (PTEN) is a bispecific lipid and protein phosphatase that negatively regulates phosphoinositide 3-kinase (PI3K)-dependent signaling pathways, accelerating the pathological progression of muscular dystrophy [170]. Research indicates that administering the PTEN inhibitor VO-OHpic trihydrate to mdx mice for three consecutive weeks significantly ameliorates the dystrophic phenotype, suggesting that targeting PTEN signalling may offer a potential therapeutic strategy for DMD [171]. Huang et al. [172] employed an emulsion solvent evaporation technique to encapsulate the PTEN inhibitor VO-OHpic within muscle-targeting peptide M12 (RRQPPRSISSH) conjugated PLGA-PEG copolymer nanoparticles, thereby constructing the functionalised nanodelivery system PLGA-PEG-M12 NPs. This system exhibits favourable physicochemical properties, including small particle size, narrow distribution, and high hydrophilicity. In vivo targeting assessments demonstrated that, compared to uncoupled PLGA-PEG NPs, PLGA-PEG-M12 NPs possess the capacity to penetrate the extracellular matrix barrier, exhibiting significantly enhanced fluorescence signals in hindlimb muscles and myoblasts. Furthermore, PLGA-PEG-M12 NPs effectively inhibited PTEN enzymatic activity without affecting protein expression levels. This induced elevated phosphorylation of downstream signalling molecules pAKT and pS6, activating the PI3K/AKT/mTOR pathway. Consequently, myoblast viability and differentiation processes were enhanced, ultimately generating new myotubes and muscle fibers. Although PLGA-PEG nanoparticles have become a well-established drug delivery platform, functional modification with the M12 muscle-homing peptide improves the local enrichment and cellular uptake efficiency of this delivery system in skeletal muscle tissue of DMD mice, thereby accelerating skeletal muscle regeneration and functional recovery in mice. **(**Table 3**)**

#### Targeted induction and fate regulation of MuSCs by nanodelivery systems

MuSCs are adult stem cells residing within skeletal muscle tissue, whose fate is precisely regulated by complex intrinsic cellular programs and niche-specific signals [173]. Activation of the Notch signaling pathway promotes the quiescence and proliferation of MuSCs [174], participating in the control of stem cell fate; however, this proliferative effect does not support subsequent myogenic differentiation. Conversely, sustained Notch signaling inhibits the expression of key transcription factors PAX7 and MYOD by interacting with the RBP-Jκ binding site upstream of Pax7, thereby suppressing satellite cell differentiation [174, 175]. Therefore, targeted inhibition of the Notch signaling pathway is considered a potential target for enhancing the directed differentiation capacity of MuSCs. Gamma-secretase inhibitors (GSIs), as potent Notch pathway inhibitors, exhibit broad recognition of gamma-secretase substrates, making them prone to off-target effects and adverse reactions, thus limiting their clinical translation [176–178]. Böcking et al. [179] developed a bone-muscle dual-layer film nanodelivery system based on mesoporous silica nanoparticles (MSNs) via spin-coating. This system loads GSIs to precisely regulate Notch signaling and induce MuSC differentiation. MSN particles coated with C2C12 myoblasts not only precisely targeted the vicinity of skeletal MuSCs and myoblasts for cellular internalization but also exhibited low immunogenicity and excellent biocompatibility. Furthermore, in vitro cellular validation yielded consistent results. The GSI-loaded nanodelivery system releases GSI to reduce expression levels of the Notch target gene Hes1 while increasing protein levels of differentiation markers such as myosin heavy chain (MHC). This indicates that the GSI-loaded nanodelivery system promotes MuSC differentiation toward myofibers by specifically inhibiting the Notch signaling pathway. **(**Table 3**)**

#### Bionic nanostructure-Induced differentiation and tissue engineering of mesenchymal stem cells into myoblasts

VML following trauma or tumor surgery overwhelms the endogenous self-repair mechanisms of skeletal muscle, preventing full restoration of its regenerative and reparative capacity [180]. Skeletal muscle tissue engineering (SMTE) is considered a promising strategy for treating volumetric muscle injury and has garnered significant attention in recent decades [181–183]. The major advantage of SMTE lies in its ability to construct a three-dimensional engineered microenvironment with specific functional properties by regulating the composition, mechanical properties, microstructure, and bioactivity of scaffold materials. Its basic strategy is to use three-dimensional scaffolds to mimic part of the structural and functional characteristics of the natural extracellular matrix and, in combination with modern biomanufacturing technologies, to integrate cells, growth factors, drugs, or other bioactive components into the scaffold system. This approach supports myoblast adhesion, oriented alignment, myogenic differentiation, and nascent myofiber formation, thereby inducing the formation of new muscle to replace damaged or lost skeletal muscle tissue [184, 185]. Therefore, functionalized three-dimensional nanoscale scaffolds should be regarded as a key engineering platform in the process of VML repair, creating favorable conditions for the restoration of skeletal muscle structure and function.

Although MuSCs support muscle self-renewal and regeneration, their in vitro expansion faces challenges such as replicative senescence and progressive loss of differentiation potential across passages, severely limiting their application in treating VML [186–188]. Mesenchymal stem cells (MSCs), multipotent stem cells derived from bone marrow, are frequently utilized in clinical autologous stem cell transplantation due to their remarkable expansion and differentiation potential, as well as their ease of acquisition [189, 190]. MSCs also represent a promising alternative cellular source to MuSCs. Despite their limited myogenic potential, MSCs can differentiate into the myogenic lineage through the expression of muscle-specific markers [191]. Previous studies demonstrated that co-culturing MSCs with the myoblast cell line C2C12 effectively promotes cell fusion between the two cell types and enhances the muscle regeneration process [191]. This may result from MSCs secreting growth factors such as basic fibroblast growth factor (bFGF), hepatocyte growth factor (HGF), or insulin-like growth factor 1 (IGF-1) [192, 193]. Studies have demonstrated that bFGF secreted by MSCs not only promotes myogenic differentiation but also enhances MSC self-renewal [194]. However, the effects of HGF and IGF-1 on the differentiation of MSCs and myoblasts remain unclear.

Witt et al. [195] utilized electrospinning technology to construct a PCL-collagen I nano-bionic scaffold with a parallel arrangement structure. MSCs and myoblasts were then seeded onto its surface to compare cellular differentiation on a two-dimensional scaffold. Results revealed that 3D culture (particularly in collagen-containing composite gels) significantly enhanced the expression of myocyte enhancer factor 2 (MEF2) and alpha-actinin-2 (ACTN2) compared to 2D culture. Following exogenous induction with HGF and IGF-1, MEF2 and ACTN2 expression was further upregulated in the co-culture group compared to the monoculture group. This suggests these factors synergistically promote MSC and myoblast differentiation, where differentiation markers produced by MSCs further enhance myogenic differentiation, yielding a synergistic “1 + 1>2” effect. Notably, this myogenic differentiation effect does not entirely depend on exogenous growth factor addition, but rather stems from the inherent potential of MSCs themselves to regulate myogenic differentiation fate.

**Fig. 3:**
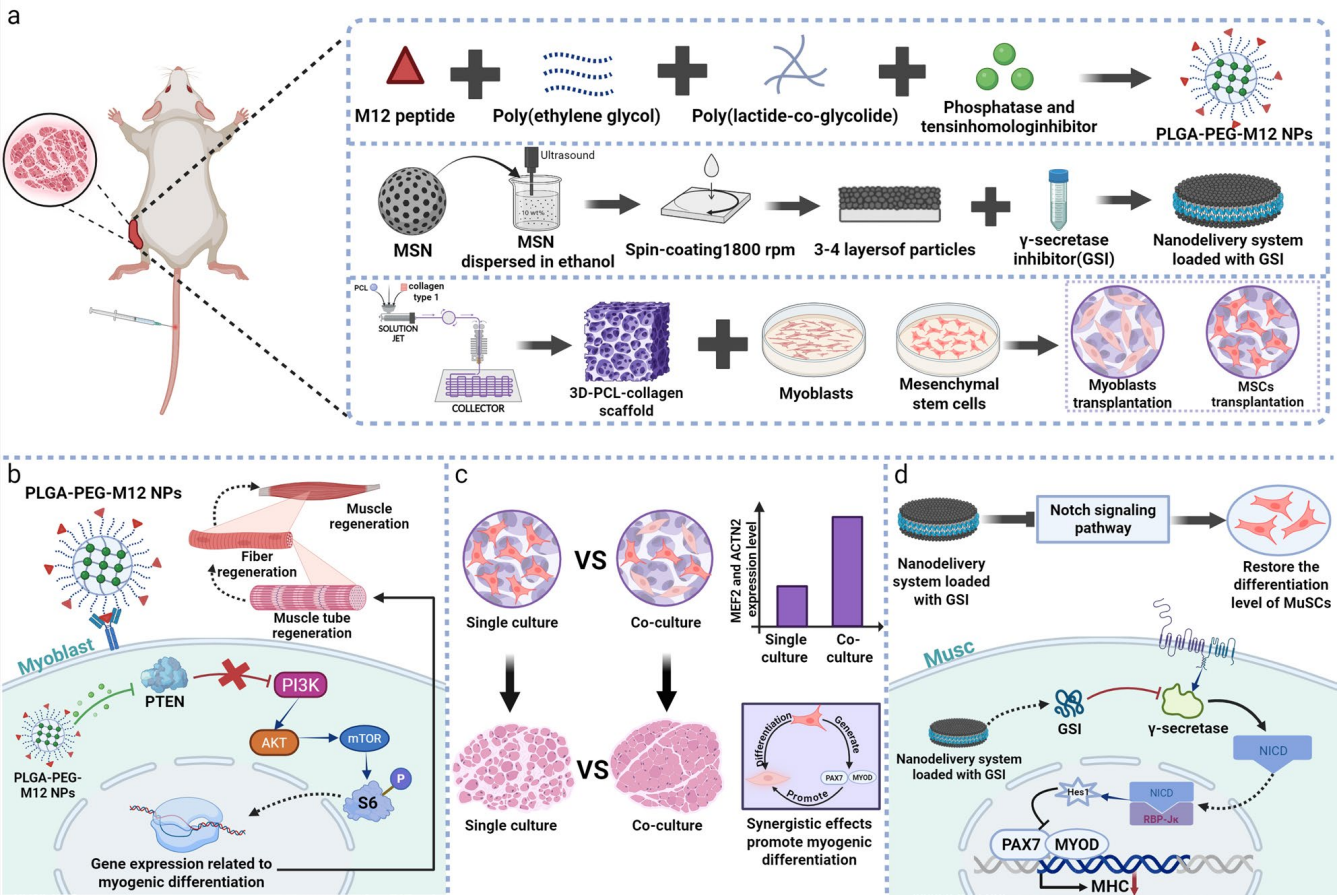
Nanotechnology-based strategies for precise induction of skeletal myocyte differentiation. (**a**) Schematic illustration of the fabrication strategies for three nanostructured systems: PLGA-PEG-M12 NPs, MSNs loaded with GSI, and a 3D-printed PCL-collagen composite scaffold. (**b)** PLGA-PEG-M12 NPs enhance myoblasts activity and differentiation by inhibiting PTEN enzymatic activity, thereby reactivating the PI3K/AKT/mTOR signaling pathway. (**c**) Co-culture of myoblasts and MSCs on the surface of the 3D-printed PCL-collagen scaffold synergistically promotes myogenic differentiation. (**d**) MSNs loaded with GSI facilitate MuSCs differentiation through significant inhibition of the Notch signaling pathway

Therefore, the PCL-collagen I nano-bionic scaffold provides a highly promising scaffold platform for VML repair. The combination of PCL and collagen provides strength, elasticity, and compliance, which are crucial for functional tissue formation. Moreover, it mimics the natural skeletal muscle structure and guides the oriented parallel alignment of MSCs and myoblasts. Induction by exogenous drugs (such as HGF and IGF-1) can synergistically promote myogenic differentiation and myofibril production, thereby accelerating the tissue repair process of VML. **(**Table 3**)** (Fig. 3).

**Table 3:** Nanotechnology-based strategies for precise induction of skeletal muscle cell differentiation

Materials	Cargo	Experimental model	Targeting Pathway	Index	Main outcome	Tissue-specific targeting	Non-specific accumulation	Cytotoxicity	Ref. No.
PLGA-PEG-M12 NPs	VO-OHpic	C2C12 myoblasts/ human skeletal myoblasts/ mdx mice	PI3K/AKT/mTOR	pAKT↑, pS6↑	Enhances nanoparticle targeting efficiency and promotes myogenic differentiation in mdx mice	High accumulation in the quadriceps, gastrocnemius and diaphragm	Minimal non-specific accumulation in the liver and kidneys	Non-toxic	^[172]^
Bilayer spin-coated MSN films (200 nm)	GSI	MSCs/ C2C12 myoblasts	Notch	Hes1↓, MHC↑	Promotes stem cell differentiation and enhances myofiber-like formation	Not reported	Not reported	Non-toxic	^[179]^
Parallel-aligned electrospun PCL-collagen-I nanofibers	Primary rat MSCs and myoblasts	Not reported	Not reported	MEF2↑, ACTN2↑, MyHC2↑	Synergistically promotes myogenic differentiation and myofiber formation	Not reported	Not reported	Not reported	^[195]^

### Nanotechnology regulation of local inflammation and ROS

Inflammatory responses and dysregulated ROS homeostasis are important microenvironmental factors involved in impaired skeletal muscle injury repair, degenerative changes, and the progression of various skeletal muscle diseases [196]. Moderate inflammatory responses and low-level ROS signaling can participate in immune cell recruitment, clearance of necrotic tissue, activation of muscle satellite cells, and tissue repair and regeneration [155, 197]. However, persistent inflammation or excessive ROS accumulation can promote the release of pro-inflammatory factors, mitochondrial dysfunction, protein degradation, and impaired myogenic differentiation, thereby hindering tissue repair and weakening functional recovery [198]. Therefore, in the context of skeletal muscle injury and disease, moderate and spatiotemporally selective regulation of local inflammatory responses and oxidative stress has become an important intervention direction for improving the pathological microenvironment of skeletal muscle.

#### Nanodelivery systems alleviating localized skeletal muscle inflammation

Targeted delivery of anti-inflammatory drugs via nanocarriers represents the most direct approach to reducing inflammation in skeletal muscle tissue. Xie et al. [199]employed strategies such as cell membrane coating and receptor-ligand interactions to prepare mimetic curcumin (Cur) nanoparticles (M12MNC) by encapsulating Cur within muscle-homing peptide (M12)-modified skeletal muscle cell membranes for treating age-related skeletal muscle atrophy. Cur, a polyphenolic compound derived from turmeric, plays a crucial role in regulating immune responses and tissue metabolism [200]. Cur protects muscle health by promoting protein synthesis, suppressing inflammation, and mitigating age-related effects [201, 202]. Studies revealed that M12MNC exhibits excellent biocompatibility and can specifically accumulate in aged skeletal muscle. M12MNCs reduced α-syn expression and promoted proliferation and differentiation in aged C2C12 cells. Following M12MNC treatment, aged mice exhibited significantly decreased expression of pro-inflammatory markers IL-6, TNF-α, and α-syn, along with markedly increased expression of the anti-inflammatory marker IL-10. Additionally, significant improvements were observed in locomotor function and skeletal muscle metabolism. Further mechanistic studies revealed that M12MNCs improve skeletal muscle dysfunction in aged mice by modulating the SphK1/Spns2/S1PR2 axis and inflammatory levels. Beyond drug delivery, inflammatory signaling molecules are also utilized to regulate the inflammatory microenvironment of skeletal muscle. Raimondo et al. [203] conjugated IL-4 with surface-polyethylene glycol-modified gold nanoparticles (AuNPs) via trehalose to prepare the IL-4 nanodelivery system (PA4). IL-4, an anti-inflammatory cytokine, induces type 2 immune responses by stimulating M2 macrophage polarization and CD4 + T helper cell phenotypes [204]. A single PA4 treatment increased regulatory T cells (Tregs) by 50% in mdx mouse muscles, quadrupling muscle fiber cross-sectional area, contractile force, and contraction velocity. By specifically modulating the local immune microenvironment, PA4 promotes Treg infiltration and functional activation, significantly improving muscle regeneration and functional recovery under chronic inflammation. **(**Table 4**)**

#### Nanodelivery systems modulate local oxidative stress in skeletal muscle

Furthermore, targeted delivery of mitochondrial modulators is equally crucial for alleviating muscle atrophy and dysfunction. Amyotrophic lateral sclerosis (ALS), a progressive neurodegenerative disease affecting motor neurons, represents a key pathogenic mechanism driving early skeletal muscle atrophy [205]. Furthermore, cellular bioenergetic defects related to mitochondrial structure and function have been observed in skeletal muscle from both sporadic ALS (sALS) and familial ALS (fALS) patients [206]. Mitochondrial abnormalities lead to excessive ROS production, exacerbating mitochondrial dysfunction and accelerating muscle atrophy [207]. However, no effective therapeutic strategies currently exist for ALS, making targeting cellular mitochondrial function a potential therapeutic approach. Malacarne et al. [208] successfully prepared FM19G11-loaded poly (lactic-co-glycolic acid) (PLGA) nanoparticles via solvent evaporation. This nanomaterial system demonstrated favorable hydrodynamic properties and encapsulation efficiency. It was employed to modulate energy metabolism and ROS levels in myoblasts from ALS mice, thereby enhancing myoblast differentiation and regeneration processes. FM19G11, as a novel small-molecule metabolic modulator, improves oxidative stress states and restores cellular energy homeostasis by inducing mild mitochondrial uncoupling. In G93A-SOD1 myoblasts treated with FM19G11-NPs, mRNA expression levels of key PI3K/AKT signaling pathway factors Akt1 and Akt3 were significantly upregulated, indicating activation of cell proliferation and activation pathways. Additionally, expression of muscle differentiation and maturation-related factors Mef2c and Ucp2 was significantly elevated, while ROS production was markedly reduced in myoblasts at the late disease stage (18 weeks). Morphological analysis of the mitochondrial network revealed significantly reduced mitochondrial area and network complexity in the FM19G11-NPs group, indicating improved mitochondrial dynamics. Thus, FM19G11 delivered via nanocarriers significantly enhances energy metabolism, antioxidant capacity, and mitochondrial function in ALS myoblasts, providing new theoretical support for developing regenerative strategies targeting skeletal muscle disease in ALS. **(**Table 4**)**

#### Synergistic strategy of engineered nanozymes for regulating local oxidative stress and regeneration in skeletal muscle

**Fig. 4:**
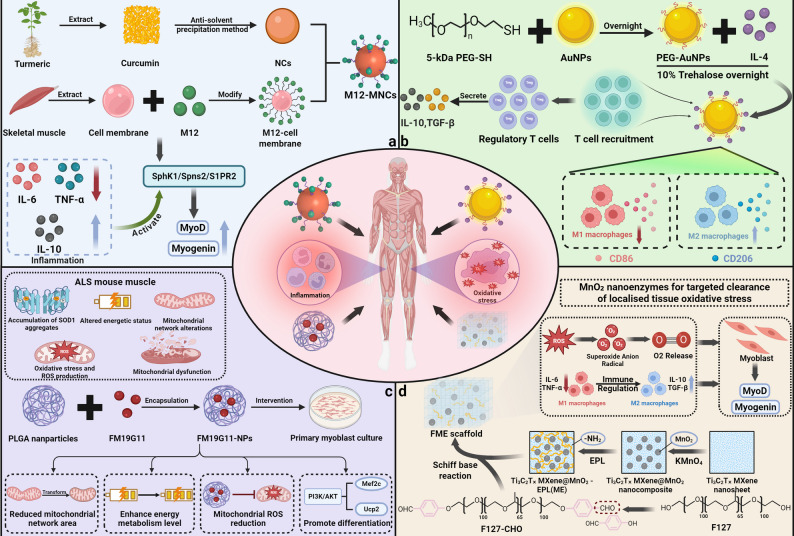
Modulation of local inflammation and ROS by nano-therapeutics. (**a**) Schematic diagram illustrating the preparation of M12-MNCs and their role in ameliorating skeletal muscle inflammation via the SphK1/Spns2/S1PR2 axis. (**b**) PEGylated AuNPs loaded with IL-4 orchestrate a type 2 immune milieu by recruiting T cells, stimulating M2 macrophage polarization, and promoting CD4 + helper T cell responses. (**c**) Pathological features of skeletal muscle in ALS mice; schematic illustration of FM19G11-NP preparation and its role in promoting myogenic differentiation by enhancing myoblast energy metabolism, antioxidant capacity, and mitochondrial function. (**d**) Fabrication process of the FME scaffold and its mechanism for skeletal muscle repair, mediated through the reduction of local ROS levels and inflammation, thereby enabling the coordinated restoration of muscle structure and function

To achieve the synergistic effects of eliminating cellular ROS and enhancing myogenic differentiation, Zheng et al. [163]prepared Ti₃C₂Tₓ MXene@MnO₂ nanocomposites by integrating MnO₂ nanozymes with Ti₃C₂Tₓ MXene nanosheets. These nanocomposites were further crosslinked with aldehyde-functionalized Pluronic F127 through Schiff-base reactions to construct an injectable, self-healing, adhesive multifunctional hydrogel scaffold (FME). FME successfully overcomes issues such as uneven material distribution, poor cell adhesion, and short retention time. It aims to treat VML by regulating myogenic differentiation, tissue inflammation and oxidative stress, as well as hypoxic microenvironments. Results demonstrate that FME significantly enhances the relative expression of MHC and myogenic genes (MyoD and MyoG) through exogenous electrical signal transduction, thereby boosting C2C12 cell proliferation, differentiation, and myotube formation capacity. Furthermore, MnO₂ nanozyme attachment targeted intracellular ROS clearance and induced macrophage phenotype shift from pro-inflammatory M1 to anti-inflammatory M2, effectively remodeling the pathological microenvironment at the injury site. Implantation of FME in a rat tibialis anterior muscle defect model significantly promotes new myofibril and vascular formation, tissue structural remodeling, and reduces local ROS levels and the expression of pro-inflammatory factors (such as IL-6 and TNF-α), ultimately achieving synergistic restoration of skeletal muscle structure and function. Therefore, the combined application of nanozymes and multifunctional scaffolds provides a multidimensional synergistic therapeutic strategy for improving the local microenvironment and regeneration of skeletal muscle. **(**Table 4**)** (Fig. 4).

**Table 4:** Nanotechnology-based strategies for modulating inflammatory and oxidative microenvironments in skeletal muscle

Materials	Cargo	Experimental model	Targeting Pathway	Index	Main outcome	Tissue-specific targeting	Non-specific accumulation	Cytotoxicity	Ref. No.
M12MNCs	Cur	D-gal-induced senescent C2C12 cells/ aging mice	SphK1/ Spns2/ S1PR2 axis	IL-6↓, TNF-α↓, IL-10↑, MyoD↑,myogenin↑, myostatin↓, α-syn↓	Alleviates age-related skeletal muscle inflammation and promotes skeletal muscle regeneration	High enrichment of gastrocnemius muscle and C2C12 cells	Less non-specific accumulation in the liver and kidneys	low cytotoxicity under the tested conditions	^[199]^
AuNPs	IL-4	MDX mice with micro-injuries to the tibialis anterior muscle	Not reported	Treg ratio↑, contraction force↑, contraction velocity↑	Promotes the remodelling of the immune microenvironment and skeletal muscle regeneration in MDX mice	High accumulation at the site of the injured tibialis anterior muscle	Not reported	Not reported	^[203]^
PLGA	FM19G11	Primary myoblasts from ALS mice	PI3K/ AKT	Akt1↑, Akt3↑, Mef2c↑, Ucp2↑, ROS↓	Significantly improves energy metabolism, antioxidant capacity and mitochondrial function in ALS myoblasts	Not reported	Not reported	Low cytotoxicity under tested conditions	^[208]^
FME	None	C2C12 myoblasts/ RAW264.7 macrophages under H2O2 stimulation/ rat tibialis anterior muscle defect model	Not reported	MHC↑, MyoD↑, MyoG↑, ROS↓, Arg-1↑, iNOS↓, IL-6↓, TNF-α↓, IL-10↑, TGF-β↑, CD31↑	Reduces oxidative stress and the expression of pro-inflammatory factors, ultimately promoting myogenic differentiation and myofiber formation	Not reported	Not reported	Good	^[163]^

### Nanotechnology against skeletal muscle infection and rhabdomyosarcoma

Modern medicine faces two major challenges in the treatment of infection and tumors. The first is the increasingly serious problem of antibiotic resistance, and the second is the difficulty of achieving efficient targeted tumor therapy [209–211]. Under pathological conditions such as trauma, surgery, immunodeficiency, and tumor infiltration, skeletal muscle is susceptible to bacterial and fungal infections as well as tumor cell invasion, thereby requiring the exploration of more locally targeted therapeutic strategies [57, 212]. To address the limitations of conventional therapies in local drug enrichment, targeted delivery, and control of toxic side effects, nanoparticles have become a promising engineered auxiliary platform for antibacterial and antitumor therapy, owing to their tunable physicochemical properties and ease of functional modification. In this section, we discuss exogenous strategies based on nanotechnology for combating skeletal muscle infection and myogenic malignancies.

#### Multifunctional nanoplatforms against skeletal muscle infections

Nanomaterials serving as antimicrobial scaffolds and carriers for antimicrobial agents ensure in vivo safety and provide direct, effective strategies for skeletal muscle tissue engineering applications. Ge et al. [213] developed a novel approach by incorporating ultramicroscopic gold@ polyethyleneimine nanoparticles (AuNPs-PEI) and polydopamine nanoparticles (PDA NPs) with alkyldoped Pluronic F127 (F127-CHO) to prepare an injectable, conductive, antioxidant, and antibacterial multifunctional nanocomposite hydrogel scaffold (FPAu) designed to promote the structural and functional integrated regeneration of VML. In vitro cell experiments demonstrated that FPAu exhibits significant antimicrobial properties, achieving up to 75% bactericidal rates against Escherichia coli and Staphylococcus aureus. It also significantly upregulates myogenic factor expression (MyoD, MyoG, TnN1) and MHC expression, promoting myotube formation, elongation, and maturation. Implantation of FPAu in a rat tibialis anterior muscle defect model increased the number of central nucleus myofibers while enhancing the ultimate tensile strength and electrically stimulated contraction function of regenerated muscle, efficiently achieving structural and functional regeneration of injured skeletal muscle. Furthermore, surface modifiers and coating technologies represent effective strategies against such infections [214]. Touseef Anma et al. [215] physically blended bio-derived hydroxyapatite (HAP) with Nigella sativa essential oil to form N. sativa-coated HAP nanoscaffolds for skeletal muscle tissue engineering aimed at the treatment of muscle atrophy and related disorders. Aromatic leaf essential oils exhibit notable antibacterial activity and can effectively inhibit or eliminate bacteria by disrupting microbial structure and function. Experimental results demonstrated that the resulting nanoscaffold possessed favorable crystalline properties and biocompatibility, and not only significantly promoted the proliferation and differentiation of C2C12 myoblasts into multinucleated myotubes, but also markedly inhibited the activity of Staphylococcus aureus. This property suggests its potential application in combating muscle atrophy associated with skeletal muscle infection. (Table 5).

#### Precision engineered nanodelivery systems for targeted treatment of rhabdomyosarcoma

For malignant lesions such as RMS that occur in skeletal muscle or adjacent muscular tissues, the value of nanoplatforms is mainly reflected in their targeted optimization of the limitations of conventional therapies, including improving drug delivery efficiency within the lesion, reducing off-target organ distribution, enhancing effective intracellular uptake, and minimizing collateral damage to the surrounding normal skeletal muscle tissue as much as possible [216]. On this basis, effective control of RMS involves not only reduction of tumor burden, but also the restoration and improvement of local skeletal muscle structural integrity and physiological function [217]. It has been reported that high expression of sialic acid on the surface of tumor cells is closely associated with malignant biological behaviors such as invasion and metastasis [218]. Therefore, the development of nanodelivery systems capable of actively targeting localized RMS, utilizing ligand-binding or RMS cell surface recognition mechanisms, holds promise for achieving selective recognition of RMS lesions [219]. Moshe et al. [220]synthesized an amphiphilic graft copolymer, PVA-g-PMMA (PVA-PMMA17), by free-radical graft copolymerization, and used PVA-PMMA17 to encapsulate the dasatinib kinase inhibitor in order to block RMS proliferation. In addition, the researchers functionalized the surface of the nanoparticles with boronic acid through non-covalent crosslinking, with the aim of actively targeting pediatric RMS cells with high sialic acid expression. The results showed that the boronic acid-modified nanoparticles not only exhibited strong targeting ability, but also enhanced cellular uptake and penetration, and significantly improved anticancer activity in two-dimensional and three-dimensional models of pediatric rhabdomyosarcoma. Further in vivo validation demonstrated that, compared with the unmodified group, intravenously injected boronic acid-modified nanoparticles achieved specific accumulation in a mouse model bearing RMS cell xenografts, while significantly reducing distribution in non-target organs such as the liver. Therefore, based on the glycosylation characteristics of the RMS cell surface, this study developed an active targeted delivery strategy by exploiting the boronic acid–sialic acid interaction, which may enhance local drug accumulation while reducing exposure in major clearance organs, thereby providing a technical basis for reducing toxicity and improving therapeutic efficacy in RMS treatment (Table 5).

**Table 5:** Nanotechnology-based antimicrobial and anti-rhabdomyosarcoma strategies

Materials	Cargo	Experimental model	Targeting Pathway	Index	Main outcome	Tissue-specific targeting	Non-specific accumulation	Cytotoxicity	Ref. No.
FPAu hydrogel scaffold	None	C2C12 myoblasts/ rat tibialis anterior muscle defect model	Not reported	MHC↑, MyoD↑, MyoG↑, Tnnt-1↑, E. coli activity↓, S. aureus activity↓	Promotes C2C12 myogenic differentiation and myotube formation, as well as the restoration of skeletal muscle biomechanics and electromechanical function	Not reported	Not reported	Good	^[213]^
HAP nanoscaffolds coated with N. sativa	None	C2C12 myoblasts	Not reported	S. aureus activity↓	Enhances the proliferation and differentiation of C2C12 myoblasts	Not reported	Not reported	Good	^[215]^
Boronic acid-conjugated PVA-g-PMMA 17 NPs	Dasatinib	Rh30 rhabdomyosarcoma 2D/3D models/ subcutaneous Rh30 xenograft mice	Not reported	RMS activity↓	Reduces tumour cell proliferation and damage to the tissues surrounding skeletal muscle	RMS expressing sialic acid in skeletal muscle	Minimal non-specific accumulation in the liver	Low cytotoxicity under tested conditions	^[220]^
glucosylated hybrid aTiO2/PEO-PPO NPs	None	Rh30 2D cells/ Rh30 spheroids/ subcutaneous Rh30 xenograft mice	Not reported	ROS↑, ATP↓	Exacerbates apoptosis in RMS cells, limits their proliferative capacity, and prolongs survival in mice.	RMS with high expression of glucose transporters in skeletal muscle	Minimal non-specific accumulation in the liver	Low cytotoxicity under tested conditions	^[221]^

**Fig. 5:**
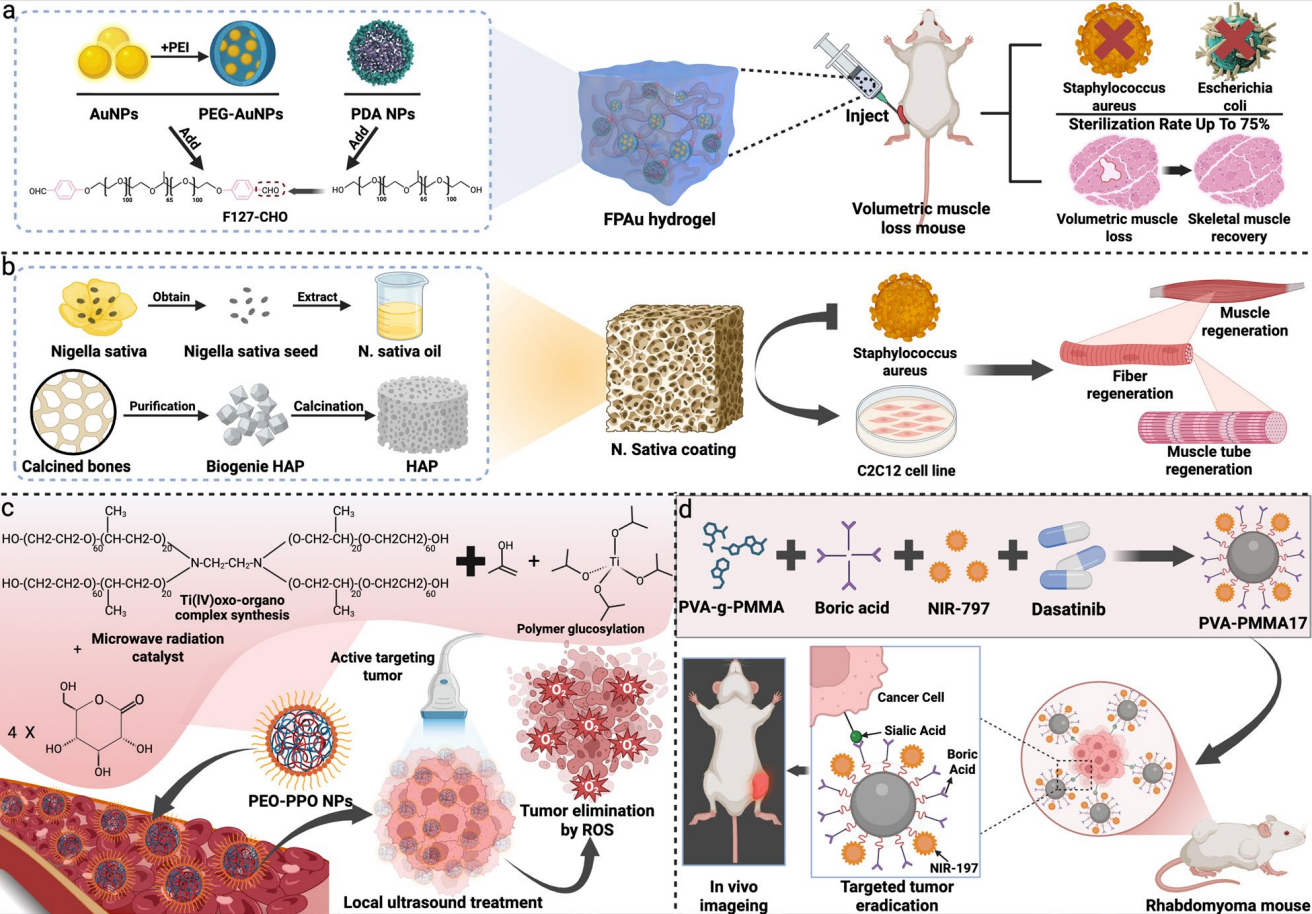
Nanomaterial-enabled therapeutics against skeletal muscle infections and rhabdomyosarcoma. (**a**) Schematic illustration of the FPAu hydrogel fabrication process. The resulting scaffold demonstrates potent antibacterial efficacy and enhances the regeneration of VML. (**b**) Fabrication process of the N. sativa-coated HAP nanoscaffold, which exhibits robust activity against Staphylococcus aureus for the treatment of skeletal muscle infections. (**c**) Illustration of the PEO-PPO NPs synthesis. These nanoparticles leverage sonodynamic targeting to initiate a Fenton-like reaction, enabling potent induction of ROS and subsequent apoptosis of tumor cells. (**d**) Preparation scheme of the PVA-PMMA17 nanomaterial. Boronic acid-functionalized PVA-PMMA17 exhibits active tumor-targeting capability, thereby augmenting its antitumor efficacy.

Apart from active targeting strategies, stimulus-responsive nanoplatforms have also provided a novel approach for precise local intervention in RMS treatment. Zlotver et al. [221] successfully developed a glucose-modified hybrid TiO2/PEO-PPO NP system with active targeting capabilities. This system uses amorphous TiO2 as the sonodynamic core, with surface stabilization provided by the amphiphilic block copolymer PEO-PPO, while glycosylation of the terminal PEO moiety enables selective recognition of tumor cells with high glucose transporter expression. In an in vitro Rh30 cell line model, glycosylated nanoparticles markedly enhanced cellular internalization by RMS cells and, upon activation by exogenous ultrasound, effectively induced reactive oxygen species generation through a Fenton-like reaction, thereby augmenting the sonodynamic cytotoxic effect. In a Rh30 skeletal muscle xenograft mouse model, this system exhibited excellent tumor-targeting and accumulation capacity, significantly suppressed tumor growth, and prolonged survival. Histological analysis further confirmed increased apoptosis of RMS cells, restricted proliferative capacity, and reduced ATP production in the treatment group. These results suggest that this nanosystem can significantly improve the spatiotemporal controllability of the intervention, thereby enhancing intralesional efficacy while minimizing nonspecific damage to the surrounding normal skeletal muscle tissue to the greatest extent possible, so as to preserve its structural and functional integrity (Fig. 5).

### Nanomaterial-supported tissue engineering for vascular network reconstruction

Skeletal muscle relies not only on soft tissues for mechanical support but also on a branched vascular network to facilitate tissue metabolism and biological functions [82]. Peripheral arterial disease causes severe lower limb blood flow insufficiency, leading to skeletal muscle atrophy, fat infiltration, fibrosis, and accompanying metabolic and cellular dysfunction [222]. Therefore, delivering exogenous growth factors to regenerate functional microvascular systems may be a key therapeutic strategy for ischemic muscle atrophy. Said et al. [223] employed electrospinning to fabricate biodegradable poly(ester amide) (PEA) nanofiber membranes loaded with fibroblast growth factor 9 (FGF9), aiming to enhance microvascular remodeling in ischemic skeletal muscle. In a chick chorioallantoic membrane (CAM) model, FGF9-loaded PEA nanofiber membranes significantly increased the density and functionality of perfused microvascular networks. In a mouse hindlimb ischemia model, following implantation of an FGF9-loaded PEA fiber membrane onto the surface of the tibialis anterior muscle, FGF9 was released into the local muscle tissue, resulting in a certain degree of factor distribution in the superficial to mid-layer muscle tissue. Notably, FGF9 treatment did not significantly increase the density of CD31-positive microvessels, but instead markedly enhanced the coverage of nascent microvessels by SM α-actin-positive vessels and PDGFRβ-positive mural cells. This finding suggests that its primary effect was not simply to induce more extensive angiogenesis, but rather to promote the maturation, stabilization, and functionalization of nascent microvessels. In addition, consistent with the above vascular maturation effect, local delivery of FGF9 was also accompanied by an increased cross-sectional area of regenerating myofibers, reduced interstitial fibrosis, and improved gait function. Taken together, in ischemic skeletal muscle repair, sustained release of vascular maturation factors may help enhance the structural stability and tissue-supportive capacity of nascent microvascular networks, thereby providing a highly translationally promising scientific basis for the treatment of ischemic skeletal muscle atrophy (Table 6).

However, for the treatment of VML, the use of reparative cell-loaded engineered scaffolds to promote angiogenesis or vascular maturation does not equate to the establishment of sufficient blood perfusion during the early stage after transplantation [224]. Since VML is usually accompanied by disruption of the pre-existing vascular bed and local ischemia, transplanted cells are susceptible to hypoxia and insufficient nutrient supply before host vascular ingrowth and effective perfusion are established [225]. Therefore, scaffold design for VML needs to further focus on the temporal relationship between angiogenesis and blood-flow restoration, so as to avoid irreversible damage to transplanted cells before functional perfusion is established. In this context, the study by Nakayama et al. [226] provides important evidence for the application of oriented nanotopographical structures and pre-endothelialization strategies in VML repair. This study exploited the pH-dependent nature of collagen fiber formation and shear-induced alignment effects to construct parallel-oriented collagen nanofiber scaffolds, and co-cultured C2C12 myoblasts with human microvascular endothelial cells to form endothelialized oriented engineered skeletal muscle tissue. Compared with randomly oriented scaffolds, this system more effectively induced myotube alignment, elongation, and maturation along the scaffold direction in vitro, and showed more pronounced MHC striation, synchronized contractile ability, and enhanced secretion of angiogenesis-related factors such as VEGF-A, angiogenin, and IGFBP-3. These results indicate that oriented scaffolds do not merely provide passive structural support, but instead facilitate the formation of organized tissue-engineered constructs with myogenic maturation and vascular-support potential before implantation through nanotopographical guidance and endothelial cell participation (Table 6).

**Table 6:** Nanotechnology-based strategies for skeletal muscle vascular integration and neuromuscular junction reconstruction

Materials	Cargo	Experimental model	Targeting Pathway	Index	Main outcome	Tissue-specific targeting	Non-specific accumulation	Cytotoxicity	Ref. No.
PEA fiber mats	FGF9	CAM model/ hindlimb ischemia model in mice	Not reported	SM α-actin↑, PDGFRβ↑	Enhances microvascular remodelling, increases endothelial cell coverage, and reduces vascular fibrosis	Midzone of ischemic skeletal muscle	Not reported	Not reported	^[223]^
Parallel-oriented collagen nanofiber scaffold	Murine myoblasts and human microvascular endothelial cells	Murine tibialis anterior VML model	Not reported	MHC↑, VEGF-A↑, CD31↑, angiogenin↑, IGFBP-3↑	Promotes microvascular regeneration and vascular integration, accelerating the structural and functional repair of skeletal muscle	Not reported	Not reported	Not reported	^[226]^
Pre-innervated aligned PCL nanofiber sheets	C2C12 myoblasts and rat spinal motor neurons	rat tibialis anterior VML model	Not reported	synaptophysin↑, α-bungarotoxin↑, Pax7↑, CD31↑, SMA↑, AchR clusters↑	Enhances muscle cell fusion and NMJ reconstruction	Not reported	Not reported	Not reported	^[231]^
Ti3C2Tx MXene/adECM conductive hydrogel	Rat L6 myoblasts and PC12 neurons	rat tibialis anterior VML model	Not reported	Cacna1a↑, Cacna1s↑, Chrna1↑, Chrnb1↑, Ca²⁺↑, MyoD1↑, Myf5↑, nAChR clusters↑	Promotes axonal growth, NMJ reconstruction and Ca²⁺-mediated neuromuscular function recovery.	Not reported	Accumulation of MXene residues has been observed in fascial tissue	Good	^[233]^

**Fig. 6:**
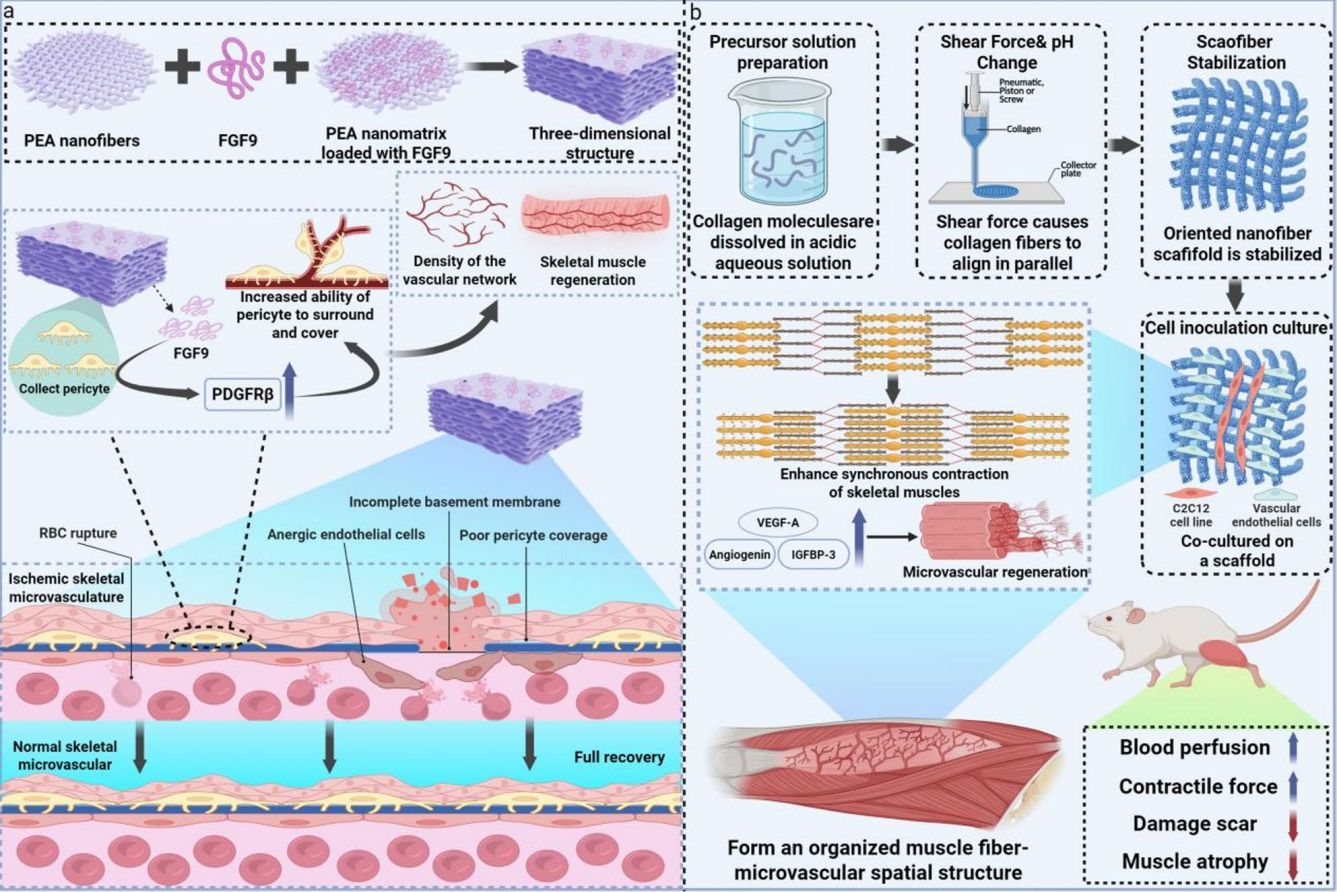
Nano-engineered scaffolds promoting angiogenesis and functional reconstruction. (**a**) Schematic of the fabrication of FGF9-loaded PEA nanofibrous membranes. The system promotes angiogenesis and pericyte recruitment by releasing FGF9 and upregulating PDGFRβ expression, enhancing their adhesion to the nascent vasculature. (**b**) Schematic of a co-culture system using C2C12 cells and human microvascular endothelial cells on aligned nanofibrous scaffolds. This setup enhances synchronous muscle contraction and pro-angiogenic factor secretion, leading to in vitro skeletal muscle regeneration

Notably, the researchers further implanted this construct into a mouse tibialis anterior VML model and examined the in vivo repair performance of the cell-loaded scaffold from the perspectives of dynamic transplanted-cell survival and functional perfusion. Bioluminescence imaging showed that the signal from transplanted cells continuously increased from days 1 to 14 after implantation and became stable from days 14 to 21, indicating that the oriented endothelialized construct could provide a more favorable microenvironment for the in vivo retention of donor cells. Meanwhile, the researchers identified perfusable vessels connected to the host circulation by CD31 and isolectin co-staining on day 21, further demonstrating that this strategy could promote the formation of a microvascular network with functional perfusion characteristics in the graft region. Taken together, this nanoscaffold can promote donor-derived myofiber formation and the parallel alignment of perfused vessels, thereby preconstructing oriented muscle–vascular integrated units and simultaneously improving transplanted-cell retention, the efficiency of vascular integration between the host and graft, and spatial tissue regeneration capacity during VML repair [226]. It should be noted that, from the perspective of reperfusion timing, although this study demonstrated the formation of perfusable vessels and improved tissue integration at the experimental endpoint after transplantation, it did not continuously analyze whether early blood-flow restoration could meet the metabolic demands of transplanted cells. Therefore, future studies should focus on whether nanoscaffold implantation can establish effective blood perfusion at an early stage, so as to improve the efficiency of tissue integration and functional recovery during skeletal muscle defect repair (Table 6) (Fig. 6).

### Nano-tissue engineering promotes neuromuscular junction remodeling and functional recovery

Skeletal muscle vascular reconstruction provides the necessary material basis for injured muscle tissue. However, whether regenerated tissue can further recover into functional muscle tissue with effective contractile and electrical conduction capacities remains highly dependent on the complete reconstruction of the neuromuscular junction (NMJ) [227]. Based on the pathological mechanisms of denervation, the major problems include the disassembly of AChR clusters, degenerative changes in the endplate structure, and insufficient reinnervation capacity, which together constitute key barriers limiting the functional recovery of skeletal muscle [228]. In response to these pathological features, nano-tissue engineering strategies have gradually shifted in recent years from simply improving the local microenvironment or promoting tissue filling toward targeted designs that regard NMJ reconstruction as a key therapeutic objective, with a particular focus on endplate structure repair and precise alignment between axons and muscle fibers, thereby accelerating neuromuscular electrophysiological maturation and functional recovery [229]. Previous studies have shown that agrin secreted by motor neurons can bind to LRP4 on the muscle fiber membrane, subsequently activate MuSK, and, with the cooperative involvement of rapsyn, promote the localization and clustering of AChRs at the endplate region, thereby providing a more stable postsynaptic receptor basis for subsequent reinnervation by nerve terminals [230]. Based on this finding, Das et al. [231] prepared aligned PCL nanofiber scaffolds using electrospinning technology and co-cultured rat spinal cord motor neurons with C2C12 myoblasts on the scaffolds, successfully constructing pre-innervated aligned engineered skeletal muscle tissue to establish a biological interaction basis between nerves and muscle in advance. In vitro experiments showed that pre-innervated co-culture significantly increased the positive co-localization of synaptophysin and α-bungarotoxin, suggesting stable structural docking between presynaptic nerve terminals and postsynaptic AChR-enriched regions, accompanied by an increased number of mature NMJs. After further implantation into a rat tibialis anterior VML model, the scaffold significantly increased the number of Pax7-positive satellite cells, the density of CD31/SMA double-positive microvessels, and the proportion and specific aggregation of AChR clusters in the peri-injury region. These findings indicate that this pre-innervated nanoscaffold can improve NMJ reconstruction after denervation and promote skeletal muscle functional recovery by synergistically promoting endplate AChR clustering, nerve terminal docking, and remodeling of the local regenerative microenvironment. Compared with the above strategies that mainly act on endplate structure and interface establishment, conductive nanocomposites are better able to provide appropriate electrophysiological stimulation to promote axonal growth and functional recovery and accelerate the functional maturation of the neuromuscular interface [232]. Jin et al. [233] uniformly introduced two-dimensional conductive Ti₃C₂Tₓ MXene nanosheets into decellularized skeletal muscle matrix and successfully constructed a composite hydrogel with both electrical conductivity and bioactivity (MXene/adECM), providing ideal structural support and electrical signal conduction conditions for neuromuscular interface reconstruction. Previous studies have shown that calcium ions (Ca²⁺), as second messengers in cells, play important roles in the metabolic homeostasis of nicotinic acetylcholine receptors (nAChRs), postsynaptic signal transduction, and NMJ maturation through calcium influx [234–236]. Experimental results showed that the MXene/adECM conductive hydrogel promoted the expression of the motor endplate-specific voltage-gated calcium channel-related genes Cacna1a (CaV2.1) and Cacna1s (CaV1.1) and enhanced Ca²⁺ influx. Further studies demonstrated that the MXene/adECM hydrogel significantly increased the expression of the nAChR subunit genes Chrna1 and Chrnb1, and fluorescence labeling confirmed a marked increase in the formation and aggregation of nAChR clusters, suggesting that the conductive microenvironment promoted NMJ reconstruction. Therefore, the MXene/adECM conductive hydrogel does not merely promote the structural formation and homeostasis of NMJs, but instead drives the transition of NMJs from morphological reconstruction toward electrophysiological functional maturation by strengthening the Ca²⁺ dynamic effects required for synaptic signal transmission (Table 6).

**Fig. 7:**
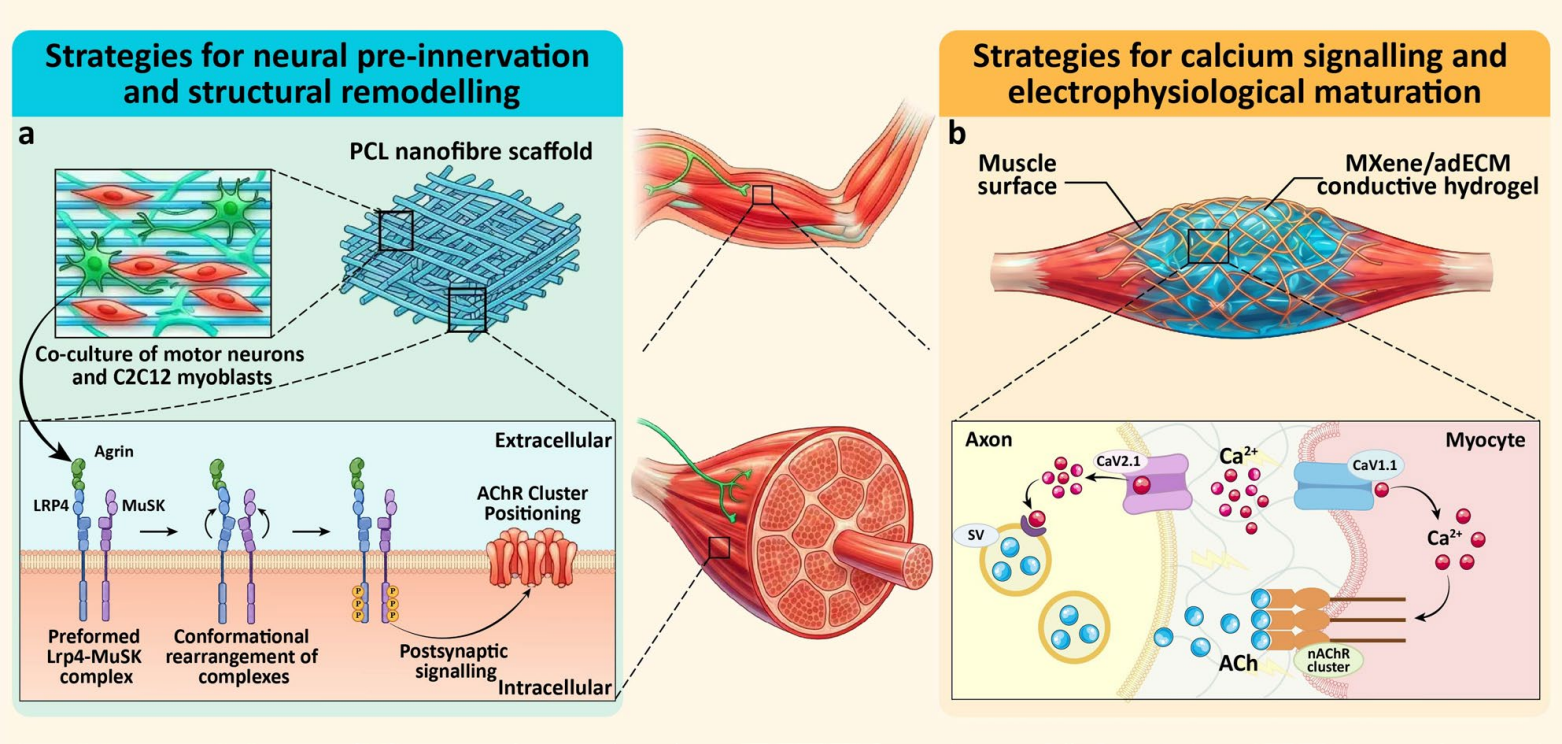
Nano-Tissue Engineering Promotes Neuromuscular Junction Remodeling and Functional Recovery. (**a**) Schematic illustration of a pre-innervated aligned PCL nanofiber scaffold. Motor neurons co-cultured on the scaffold secrete Agrin, which binds to LRP4 on the sarcolemma and activates downstream postsynaptic signaling with the involvement of MuSK, thereby promoting AChR clustering and NMJ structural reconstruction. (**b**) The conductive and bioactive MXene/adECM composite hydrogel promotes the expression of voltage-gated calcium channel-related genes and nAChR-related genes, enhances Ca^2+^ influx and nAChR clustering, and thereby facilitates NMJ reconstruction and functional maturation

In summary, in response to pathological states after skeletal muscle denervation, including destabilized AChR clustering, endplate structural degeneration, and defective reinnervation function, current skeletal muscle nano-tissue engineering mainly focuses on NMJ structural reconstruction and functional maturation as the core interventional strategy, thereby promoting the transition from tissue regeneration to functional repair. At the same time, this strategy provides new research ideas and therapeutic approaches for the treatment of VML and other related skeletal muscle diseases (Table 6) (Fig. 7).

## Challenges

### Biocompatibility and long-term safety concerns

In the biomedical field, biocompatibility is an important prerequisite for evaluating the clinical application potential of nanomaterials. Poor biocompatibility may induce cytotoxicity, abnormal immune responses, and tissue injury, thereby severely affecting cellular metabolism and the entire process of tissue repair. For example, some metallic or other inorganic nanomaterials, such as AuNPs and MXenes, still have limitations in cellular uptake, tissue transport, degradation metabolism, and systemic clearance. For local skeletal muscle therapy, whether the material itself can be effectively transported out of muscle tissue, degraded, and ultimately eliminated from the body after mediating drug delivery, catalytic regulation, or scaffold integration remains a key bottleneck affecting long-term safety [237]. Existing studies have demonstrated that the size, morphology, surface chemistry, and protein corona formation of inorganic nanomaterials can influence their renal excretion, hepatobiliary clearance, and uptake by the mononuclear phagocyte system. Among them, smaller particles or materials with degradable components may be eliminated in urine through glomerular filtration, whereas poorly degradable, larger, or phagocyte-internalized particles are more likely to undergo slow clearance or long-term retention in local tissues, draining lymph nodes, or the hepatic and splenic mononuclear phagocyte system [238, 239]. In addition, in skeletal muscle injury microenvironments such as VML, extracellular matrix deposition, insufficient local perfusion, and inflammatory-cell infiltration can restrict the transport of particles from the muscle interstitium into capillaries or the lymphatic drainage system, making inorganic nanomaterials more prone to retention in the local interstitium and within macrophages [240]. Such long-term retention may lead to persistent macrophage activation, oxidative stress, chronic inflammation, or fibrotic responses, thereby interfering with myofiber repair and functional recovery [241]. Therefore, future safety evaluation of nanomaterials should not only focus on short-term biocompatibility, but also establish a comprehensive assessment framework covering local retention, intracellular accumulation, lymphatic transport, systemic clearance, and long-term tissue toxicity, so as to accurately evaluate their clinical translational risks.

### Challenges in precise controlled release and mechanism exploration

Although nanodelivery systems and bioscaffolds have shown considerable potential in skeletal muscle repair and regeneration, their further translational application remains constrained by insufficient precision in controlled release, limited targeting specificity, and an incomplete understanding of their underlying mechanisms of action [242]. First, the release rate, release profile, and local exposure duration of therapeutic cargos remain difficult to precisely regulate, which may result in insufficient local concentrations, mismatched exposure windows, and burst release-associated adverse effects, thereby weakening their sustained regulatory effects on the injured microenvironment. Furthermore, after internalization by cells, drugs and growth factors activate downstream signaling pathways directly or indirectly, initiating cascading reactions that modulate cellular biology and promote skeletal muscle regeneration. However, the precise regulatory mechanisms and spatiotemporal dynamics of these signaling networks remain incompletely elucidated, necessitating further systematic investigation. Therefore, real-time monitoring of the release kinetics, spatial distribution, local concentrations, and metabolic clearance of drugs and growth factors is crucial for deepening our understanding of the intrinsic mechanisms of action within nanosystems and objectively evaluating their therapeutic efficacy. Currently, integrating advanced nanoimaging technologies with multimodal tracing methods (such as fluorescence imaging, radiolabeling, and mass spectrometry imaging) holds great promise for providing robust technical foundations to optimize delivery systems and achieve precision therapy [243].

### Limitations of functional biomimicry in scaffolds

The application value of engineered nanoscaffolds lies in their ability to provide structural support and local microenvironmental modulation for tissue repair by regulating material composition, microstructure, pore architecture, and spatial orientation [244]. However, skeletal muscle repair depends not only on the overall morphological design of the scaffold, but more importantly on whether it can achieve structural layering, directional alignment, and functional integration with host skeletal muscle in vivo, which remains a key issue affecting functional tissue recovery [245]. In addition, high production costs and dependence on precision manufacturing technologies also limit the scalable fabrication and clinical translation of these scaffold systems. Taking 3D-bioprinted conductive scaffolds as an example, their high production cost is not only derived from materials, fabrication processes, and equipment expenses, but is also closely associated with complex functional biomimetic requirements and precision-manufacturing demands. In VML repair, these scaffolds need to maintain myofiber-like orientation, three-dimensional pore interconnectivity, and continuous conductive pathways, and they also need to establish effective perfusion as early as possible after implantation to reduce ischemia, hypoxia, and transplanted-cell necrosis. Meanwhile, whether the conductive structure can connect with host nerves and support functional electrophysiological conduction remains an important challenge for long-term contractile recovery. To overcome these limitations, conductive-component-containing bioinks must further balance bioprinting stability, cell-loading capacity, continuous distribution of conductive components, mechanical matching, and electrical signal-transmission efficiency, which also increases the difficulty of formulation screening, printing-parameter optimization, and batch-to-batch consistency control. Therefore, the clinical translational bottleneck of 3D-bioprinted conductive scaffolds lies not only in scaffold structural design or the introduction of conductive components, but more importantly in whether a reproducible, quality-controllable, and cost-acceptable preparation system can be established while achieving structural biomimicry, continuous conductivity, early perfusion, neural integration, and long-term functional recovery in large-scale constructs.

## Outlook

### Smart sensing nanomedicine technologies

Although advancements in skeletal muscle tissue engineering have brought promising progress to regenerative medicine, future development hinges on achieving highly precise, minimally invasive, and personalized disease diagnosis and treatment. Early diagnosis of skeletal muscle disorders is crucial for slowing disease progression and improving patient quality of life. To address the limitations of traditional imaging and biopsy methods—including insufficient sensitivity, high invasiveness, and inability to provide real-time monitoring—supersurface-based nano-optical sensors demonstrate significant advantages. This technology employs subwavelength structural units to precisely modulate multidimensional optical field parameters—including amplitude, phase, and polarization state—enabling simultaneous, label-free detection of biomarker refractive index distributions, molecular vibrational spectra, and chiral signals. This facilitates highly sensitive, multiparametric sensing of target analytes [246]. Furthermore, by integrating machine learning algorithms to intelligently analyze images or intensity distributions from metasurface sensors, these devices can replace traditional spectroscopic instruments. This substitution of costly conventional spectroscopic equipment advances the development of portable, low-cost in vitro diagnostic platforms [247]. In the future, such sensing technologies integrating micro-nano photonics with artificial intelligence algorithms are expected to provide a robust key technological platform for the early diagnosis of skeletal muscle diseases, the regulation of acoustic-optical-magnetic multimodal dynamic responses, and the elucidation of deep molecular mechanisms. This will advance the development of precision diagnosis and treatment systems for skeletal muscle-related diseases.

### Microenvironment-specific response nanodelivery systems

Microenvironment-responsive nanodelivery systems provide a promising design strategy for improving controlled cargo release and local therapeutic exposure. These nanosystems mainly exploit biochemical cues associated with diseased tissues, such as local pH changes, elevated ROS levels, specific enzymatic activity, or aberrant expression of inflammatory factors, to promote the condition-dependent release or activation of therapeutic cargos, including drugs, cells, or bioactive molecules. This approach may help reduce premature release to some extent and improve local bioavailability. For example, in response to the acidic microenvironment of tumor cells, ultrasensitive pH-dependent nanodelivery systems can be designed to prolong drug release and retention, thereby providing strong synergistic therapeutic effects for combined radiotherapy and chemotherapy. In osteoporotic models, the pH of the osteoclast-mediated bone resorption microenvironment can decrease to 3–4. Dou et al. [248] developed an osteoclast-associated microenvironment-responsive delivery system by covalently conjugating pH-sensitive enzymes with nanocomposites, which showed beneficial effects in local microenvironmental regulation. In recent years, nanodelivery systems have also been explored in anti-aging research. Based on the elevated senescence-associated secretory phenotype (SASP) and ROS levels in senescent cardiomyocytes, Wu et al. [249] developed a self-assembled nanocarrier with CD9-targeting capability and ROS-responsive release properties, enabling selective clearance of senescent cells. Nevertheless, it should be emphasized that microenvironmental responsiveness is not equivalent to precise target specificity. Because local microenvironmental cues are often dynamically regulated, spatially heterogeneous within and beyond lesions, and partially overlapping with signals in non-target tissues, this strategy should be regarded as a potential approach to mitigate nonspecific exposure and optimize local release behavior, rather than as a definitive solution to off-target effects. Therefore, future studies should further integrate active targeting ligand modification, spatiotemporally controlled release design, and dynamic in vivo tracing assessment to systematically improve the delivery precision and translational feasibility of this strategy in skeletal muscle-related diseases.

### Multimodal therapy in nano-tissue engineering

Research in tissue engineering increasingly focuses on the development and application of conductive biomaterials. Inspired by the electrophysiological properties of natural cellular microenvironments, exogenous electrical stimulation plays a crucial role in regulating intracellular signaling and intercellular communication [250]. Advanced fabrication techniques such as piezoelectric electrospinning, self-assembly, and 3D printing enable the construction of three-dimensional scaffolds that combine porous structures with conductive properties. Such scaffolds not only effectively guide cell adhesion and direct differentiation, but also mimic the physicochemical and electrical microenvironment of the native ECM, thereby offering a feasible strategy to help address the shortage of transplantable organs. Furthermore, in vitro models of health or disease constructed using conductive materials hold promise for significantly enhancing the accuracy and safety of drug screening processes. With technological advancements, conductive nanobiomaterials are emerging as prime candidates for bioinks. Combined with cutting-edge 3D bioprinting techniques (such as inkjet, extrusion, and laser-assisted bioprinting), these materials enable the precise fabrication of engineered skeletal muscle tissue with contractile function, and even full-scale human muscle structures. Moreover, leveraging the unique physical properties of 3D conductive bio-scaffolds, responsive behavior to external stimuli (such as sound, light, heat, and magnetic fields) can be achieved.

## Data Availability

No datasets were generated or analysed during the current study.

## Abbreviations

TENS: Transcutaneous electrical nerve stimulation
DMD: Duchenne muscular dystrophy
MuSCs: Muscle satellite cells
NOS: Nitric oxide synthase
NO: Nitric oxide
TLRs: Toll-like receptors
UPS: Ubiquitin-proteasome system
LPS: Lipopolysaccharide
ROS: Reactive oxygen species
NOX2: NADPH oxidase 2
IARC: International Agency for Research on Cancer
RMS: Rhabdomyosarcoma
SDT: Sonodynamic therapy
PTT: Photothermal therapy
PDT: Photodynamic therapy
T2DM: Type 2 diabetes mellitus
NMJ: Neuromuscular junction
PEG: Polyethylene glycol
AChRs: Acetylcholine receptors
NMES: Neuromuscular electrical stimulation
3D bioprinting: Three-dimensional bioprinting
GelMA: Gelatin methacryloyl
PPy: Polypyrrole
PANI: Polyaniline
PCL: Polycaprolactone
OXD-like: Oxidase-like
SOD-like: Superoxide dismutase-like
CAT-like: Catalase-like
POD-like: Peroxidase-like
TPP: Triphenylphosphonium
mROS: Mitochondria-derived ROS
Pd: Palladium
CDT: Chemodynamic therapy
PTEN: Phosphatase and tensin homolog
PI3K: Phosphoinositide 3-kinase
GSIs: Gamma-secretase inhibitors
MSNs: Mesoporous silica nanoparticles
MHC: Myosin heavy chain
VML: Volumetric muscle loss
SMTE: Skeletal muscle tissue engineering
MSCs: Mesenchymal stem cells
bFGF: Basic fibroblast growth factor
HGF: Hepatocyte growth factor
IGF-1: Insulin-like growth factor 1
MEF2: Myocyte enhancer factor 2
ACTN2: Alpha-actinin-2
Cur: Curcumin
ALS: Amyotrophic lateral sclerosis
sALS: Sporadic ALS
fALS: Familial ALS
PLGA: Poly(lactic-co-glycolic acid)
PDA NPs: Polydopamine nanoparticles
F127-CHO: Alkyldoped Pluronic F127
HAP: Hydroxyapatite
FGF9: Fibroblast growth factor 9
PDGFRβ: Platelet-derived growth factor receptor β
CAM: Chorioallantoic membrane
SM α-actin: Smooth muscle α-actin
SASP: Senescence-associated secretory phenotype
ECM: Extracellular matrix
nAChRs: Nicotinic acetylcholine receptors
CaV2.1: Cacna1a
CaV1.1: Cacna1s
